# Development of a Quantitative BRET Affinity Assay for Nucleic Acid-Protein Interactions

**DOI:** 10.1371/journal.pone.0161930

**Published:** 2016-08-29

**Authors:** Timothy A. Vickers, Stanley T. Crooke

**Affiliations:** Department of Core Antisense Research, Ionis Pharmaceuticals, Inc., 2855 Gazelle Court, Carlsbad, CA, 92010, United States of America; Florida Atlantic University, UNITED STATES

## Abstract

Protein-nucleic acid interactions play a crucial role in the regulation of diverse biological processes. Elucidating the roles that protein-nucleic acid complexes play in the regulation of transcription, translation, DNA replication, repair and recombination, and RNA processing continues to be a crucial aspect of understanding of cell biology and the mechanisms of disease. In addition, proteins have been demonstrated to interact with antisense oligonucleotide therapeutics in a sequence and chemistry dependent manner, influencing ASO potency and distribution in cells and *in vivo*. While many assays have been developed to measure protein-nucleic acid interactions, many suffer from lack of throughput and sensitivity, or challenges with protein purification and scalability. In this report we present a new BRET assay for the analysis of DNA-protein interactions which makes use of an extremely bright luciferase as a tag for the binding protein, along with a long-wavelength fluorophore conjugated to the nucleic acid. The resulting assay is high throughput, sensitive, does not require protein purification, and even allows for quantitative characterization of these interactions within the biologically relevant context of whole cells.

## Introduction

Proteins interact with DNA and RNA through electrostatic interactions, hydrogen bonding, hydrophobic interactions, and base stacking [1–4]. These forces contribute in varying degrees to proteins binding in a structure and sequence specific or non-sequence specific manner [5]. Understanding how proteins interact with nucleic acids and identifying the nucleic acid sequence (and possibly structure) required to assemble these complexes are vital to understanding the role these complexes play in regulating cellular processes. Furthermore, the broad and increasing use of oligonucleotides of various types as research tools and platforms for drug discovery and development demand a more thorough understanding of how these pharmacological agents interact with various proteins and how chemical modifications, sequence and structure influence interactions with proteins. We have recently demonstrated that approximately 50 intracellular proteins interact with phosphorothioate modified antisense oligonucleotides (PS ASOs) [6]. To fully characterize interactions with all of these proteins and various mutants is currently a daunting task due to the need to generate and purify proteins to evaluate binding interactions. In addition, antibodies must frequently be generated to facilitate isolation and evaluation of proteins. Moreover, it would be ideal to be able to compare binding to the purified protein with binding to the protein of interest in the native complexes present in the cell or cell homogenates.

In this paper we report the development of a new assay that is rapid, high throughput, and provides information on protein-nucleic acid interactions that is not provided by other assays. The assay is based on a bioluminescence resonance energy transfer (BRET) assay utilizing the recently introduced Nanoluc luciferase (Nluc) [7], which has resulted in a significant improvement in the performance of BRET assays for protein/protein interactions (NanoBRET) [8–10]. Nluc is an engineered protein, which uses a novel coelenterazine derivative (furimazine) as its substrate. Its small size (19 kDa) and high physical stability result in minimal influence when tethered to other proteins. Although it is only about half the size of Rluc, it produces sustained luminescence with much greater intensity, allowing very small quantities to be accurately quantified. Moreover, its bioluminescence spectrum is narrower than Rluc, permitting better spectral discrimination with acceptor fluorophores.

We took advantage of these unique biochemical properties of NLuc to develop a BRET assay which relies on the transfer of light energy from an Nluc tagged binding protein acting as the BRET donor, to a fluorescently tagged ASO acting as the BRET acceptor. This rapid and high throughput BRET assay works with immunopurified proteins, cell homogenates, or even in intact cells, and can generate K_D_’s and relative K_D_’s for the Protein/ASO interaction. The assay also provides information on relative binding distances and orientation and does not require protein purification or denaturation, thereby supporting evaluation of native fusion protein/ASO interactions. In addition, mutant proteins can be rapidly generated and expressed to obtain information on binding domains and protein structure. In addition to interactions of proteins with modified ASOs, the assay can also be used to measure interactions between natural nucleic acids and their binding partners. We present data demonstrating the utility of the BRET assay for binding of dsDNA by transcription factors, and for measuring affinity of structured RNAs to RNA binding proteins.

## Materials and Methods

### Preparation of antisense oligonucleotides

Synthesis and purification of phosphorothioate/2’-MOE, 2’-F or S-cEt oligonucleotides was performed as described previously [11]. Standard phosphodiester deoxy and ribo oligonucleotides were obtained from Integrated DNA technologies (Coralville, Iowa). Oligonucleotides used in this work are detailed in S1 Table.

### Construction, expression, and purification of fusion proteins

NanoLuc fusion protein construction, expression, and purification were performed using the vectors pFN31K Nluc CMV-neo for amino-terminal clones and pFC32K Nluc CMV-neo for carboxy-terminal clones (Promega). Briefly, the gene of interest (GOI) was amplified using PCR primers complementary to full length cDNAs obtained from Origene. For cloning into pFN31K Nluc CMV-neo, the forward PCR primer was comprised of sequence complimentary to the sequence of the GOI following the AUG start preceded by an XhoI site for cloning in frame with NLuc, whereas the reverse primer was complementary to the sequence preceding the stop codon of the GOI followed by an EcoRI site. The PCR amplified product was then digested with XhoI and EcoRI then ligated into the pFN31K Nluc CMV-neo vector prepared with the same enzymes using standard techniques. For cloning into pFC32K Nluc CMV-neo, the forward PCR primer was comprised of sequence complimentary to the GOI sequence, including the AUG start codon preceded by a Kozak sequence and an NheI site (gctagcAGCCACC), whereas the reverse primer was complementary to the sequence, including the stop codon of the GOI followed by an XhoI site. The PCR amplified product was then digested with NheI and XhoI and ligated into the pFC32K Nluc CMV-neo vector prepared with the same enzymes. Sequences of PCR cloning primers can be found in S2 Table.

Deletion mutants for P54nrb, La, and RNAse H1 were generated by site directed mutagenesis of the fusion constructs described above using a QuikChange Lightning Site Directed Mutagenesis (SDM) Kit (Agilent Technologies) essentially as detailed previously [12]. Sequences of SDM primers can be found in S3 Table.

NLuc fusion proteins were expressed by transfecting into 6 x 10^5^ HeLa cells using Effectene transfection reagent according to the manufacturer’s protocol (Qiagen). Following a 24 hour incubation, cells were removed from the plate by trypsinization, pelleted, washed 1X with PBS, then resuspended in 250 μl Pierce IP Lysis Buffer (Thermo Scientific). Lysates were incubated 1 hour at 4°C while rotating, then debris pelleted by centrifugation at 15,000 rpm for 5 min. Fusion proteins were immunoprecipitated overnight at 4°C while rotating using 1–2 μg of antibody specific to the GOI or NLuc (Promega) (S1 Fig). The following day, 20 μl of Pierce Protein G Magnetic Beads (Thermo Scientific) were added and the incubation continued for 2 hours. Beads were washed 4X with IP Lysis Buffer and finally suspended in 250 μl 2X binding buffer (0.2 M Potassium acetate, 40 mM Tris, pH 7.5, 2 mM EDTA, .02% NP-40). Western blots were carried out as detailed previously [13].

### BRET binding affinity assay

BRET assays were performed in white 96 well plates. Alexa-linked ASOs at the indicated concentrations were incubated at room temperature for 15 min in 1X binding buffer with 10^6^ RLU/well of immunoprecipitated NLuc fusion protein or whole cell lysate. Following the incubation, NanoGlo substrate (Promega) was added at 0.1 μl/well. Readings were performed for 0.3 sec using a Glomax Discover system using450 nm/8 nm band pass for the donor filter, and 600 nm long pass for the acceptor filter. BRET was calculated as the ratio of the emission at 600/450 (fluorescent excitation emission/RLU). For competitive binding assays the Alexa-linked ASO was added at approximately the K_D_ for the particular fusion protein and the unconjugated competing ASO added at the indicated concentrations in 50 μl water. 10^6^ RLU/ well of immunoprecipitated fusion protein or whole cell lysate well was then added in 50 μl 2X binding buffer for a final volume of 100 μl. After a 15 min incubation at room temperature, substrate addition and BRET readings were carried out as detailed above.

### Permeabilized cell assay

NLuc fusion plasmids were transfected into 6 x 10^5^ HeLa cells using Effectene transfection reagent as detailed above. After 4 hours transfection, reagent was removed, cells trypsinized and then plated in 96 well white clear bottom plates at 5000/well. After an overnight incubation, growth media was removed, and cells washed 1X with PBS. Cells were then incubated in OptiMEM media + 25 μg/ml digitonin for 5 min, then Alexa-linked ASOs at indicated concentrations, followed by a 15 min incubation at 37°C. Substrate addition and BRET readings were then carried out as detailed above.

### Fluorescent polarization assay

The 20nt 3’Alexa-linked cEt gap-mer ASO was combined at 0.3 nM with unconjugated competing ASO at the indicated concentrations in 50 μl water. 50 μl of 10 nM purified HIS-tagged P54nrb protein in 2X binding buffer was then added. Following a 15 min incubation, fluorescence polarization was read using an Infinite M1000 PRO plate reader (TECAN) with an excitation wavelength of 590 ±5 nm and an emission wavelength of 620± 20 nm.

## Results

**Fig 1:**
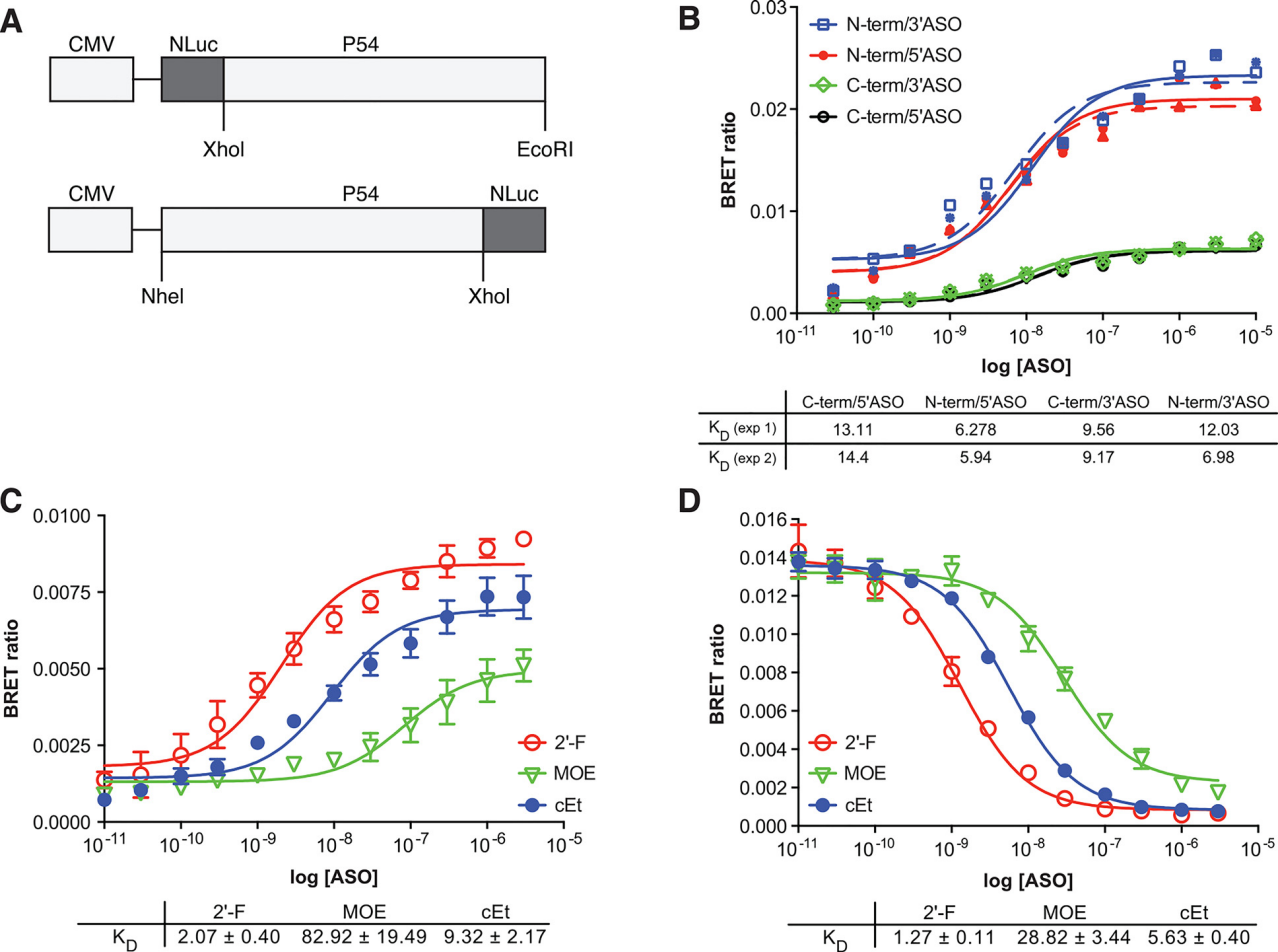
**A)** General cloning strategy for NLuc fusion proteins. P54nrb cDNA was amplified by PCR to include an N-terminal XhoI site and a C-terminal EcoRI site. The resulting cDNA was cloned in frame using the same sites in the plasmid pFN31K Nluc. For cloning into pFC32K Nluc the cDNA was amplified by PCR to include an N-terminal NheI site and a C-terminal XhoI site. Primer sequences can be found in S2 Table. Expression of all clones is driven from the CMV promoter. The resulting fusion plasmids were expressed in HeLa cells. **B)** ASO/BRET affinity for p54nrb. P54nrb/NLuc fusion proteins were immunopurified as detailed in Materials and Methods and subsequently incubated with Alexa 594 conjugated 5-10-5 cEt gap-mer ASO at concentrations ranging from 10 pM to 10 μM. BRET ratios were determined for P54nrb-NLuc (green, black) or NLuc-P54nrb (red, blue) with either a 3’ conjugated (766636) or 5’ conjugated (766635) ASO. Concentration response curves and K_D_’s (nM) for two independent experiments are shown. **C)** ASO/BRET binding affinity varies with chemistry of the 2’ modification. ASO/BRET assay was performed with P54nrb-NLuc fusion and 5’ conjugated 5-10-5 ASOs at concentrations ranging from 10 pM to 10 μM. 2’-F, red; cEt, blue; MOE, green. **D)** Relative affinities for 2’F (red), cET (blue), and MOE (green) gap-mer ASO as determined by competitive ASO binding to the NLuc/P54nrb fusion protein in the BRET assay. 10 nM 3’ Alexa conjugated cEt ASO (766636) was competed with unconjugated 5–10–5 2′-F (red), MOE (green), or cEt (blue) gap-mer ASO at concentrations from 0.1 to 1000 nM. Relative K_D_’s are shown. Data in panels C and D are mean ± SEM from 3–4 independent experiments.

It was recently reported that the Drosophila behavior/human splicing (DBHS) family proteins P54nrb (NONO), PSF (SFPQ), and PSPC1 can bind to PS ASOs, resulting in inhibition of ASO directed RNAseH1-mediated activity [14]. In these experiments, association of proteins with ASOs was determined by affinity selection of proteins bound to PS-ASO/RNA duplex. Interactions between P54nrb/PSF and single-stranded PS-ASOs are mainly influenced by the PS modification, however different 2’ moieties can further influence the binding of PS-ASOs to P54nrb [15]. To investigate interactions of modified ASOs with proteins in a more quantitative manner, we sought to develop a binding assay that was rapid, robust, and highly quantitative. We developed a BRET affinity assay in which NLuc was fused in frame to P54nrb at either the amino- (NLuc-P54nrb) or carboxy-terminus (P54nrb-NLuc) of the protein under the control of the CMV immediate early enhancer/promoter as detailed in Materials and Methods (Fig 1A). Transfection of HeLa cells with the P54nrb fusion constructs resulted in significant levels of the fusion protein as determined by western blot analysis (S1 Fig). Whole cell lysates were generated and the fusion protein immunoprecipitated using an antibody directed to P54nrb. To normalize for differences in expression levels, 10^6^ RLU/well of the immunoprecipitated fusion protein were incubated in a 96-well plate with PS-modified, 5–10–5 “gap-mer” ASOs containing 10 deoxyribonucleotides in the middle flanked at both ends by five 2′-constrained ethyl (cEt) and conjugated at either the 5’ or 3’ end with Alexa 594 (S1 Table) at concentrations ranging from 10 pM to 10 μM. NLuc substrate was then added and BRET ratios determined as detailed in Materials and Methods. The K_D_ for ASO binding was determined to be 9–14 nM for P54nrb-NLuc and 6–12 nM for NLuc-P54nrb. Interestingly, the amplitude of the BRET signal was greater with NLuc-P54nrb than P54nrb-NLuc. BRET efficiency is a measure of distance between the donor and the acceptor, which varies according to an inverse sixth power of the distance between the two molecules [16]. Therefore, the greater amplitude of signal with the NLuc-P54nrb construct may be indicative of the ASO binding nearer the amino terminus than the carboxy terminus of P54nrb. Such an interaction would be consistent with the ASO binding either of the RNA recognition motifs found near the amino terminus of the P54nrb protein [17]. These results were confirmed by evaluating ASO binding to La, a conserved component of eukaryotic ribonucleoprotein complexes that binds the 3' poly (U)-rich elements of nascent RNA polymerase III (pol III) transcripts to assist folding and maturation [18]. In addition, La has previously been shown to interact with PS ASOs [6]. Similar to P54nrb, the amplitude of the BRET signal was significantly greater for NLuc-La than the La-Nluc construct (S2A Fig), consistent with the structural organization of the La RNA binding domains nearer the amino terminus of the protein [19].

As it was previously observed that the affinity of ASOs for P54nrb is highly influenced by the 2′ modification included in the “wings” of the ASO gap-mer [14], we evaluated the effect of 2’ modifications on ASO affinity for P54nrb by BRET. Approximately 10^6^ RLU/well of the immunoprecipitated P54nrb-NLuc fusion protein were incubated in a 96-well plate with 20 nucleotide 5-10-5 cEt, 2′-fluoro (2′-F), or 2′-O-methoxyethyl (MOE) gap-mer ASOs conjugated at the 5’ end with Alexa 594 at concentrations ranging from 10 pM to 10 μM. NLuc substrate was then added and BRET ratios determined. As observed previously, 2’ modifications had a significant effect on the affinity of the ASO for P54nrb, with the 2’-F binding with a K_D_ of 2.1 nM, the cEt 9.3 nM, and the MOE 82.9 nM (Fig 1C). This is the same rank order of affinity previously observed by affinity selection. 2’modifications had a less significant effect on ASO affinity for NLuc-La protein, with the 2’-F binding with a K_D_ of 1.1 nM, the cEt 5.0 nM and the MOE 9.3 nM (S2B Fig).

We next sought to broaden the utility of the assay by eliminating the requirement that the ASO be conjugated to an ALEXA fluorophore. A competitive NLuc-P54nrb binding assay was performed in which 10 nM Alexa conjugated 2’F ASO was competed with unconjugated 5–10–5 2′-F, MOE, or cEt gap-mer ASO at concentrations from 0.1 to 1000 nM. As with direct binding, competitive binding curves revealed the same rank order in binding affinity based upon 2’ modification of the competing ASO: 2’F >cEt >MOE (Fig 1D). However, while the relative K_D_’s were similar to those observed by direct binding for the 2’F and cEt, the K_D_ for the MOE was higher in the competitive binding assay. This may result from differences in binding of the MOE ASO to the N-terminally vs C-terminally conjugated P54nrb fusion protein. K_D_’s obtained by competitive binding of NLuc-La were very similar to those obtained by direct binding and also followed the same rank order based upon the 2’ modification of the ASO (S2C Fig).

**Fig 2:**
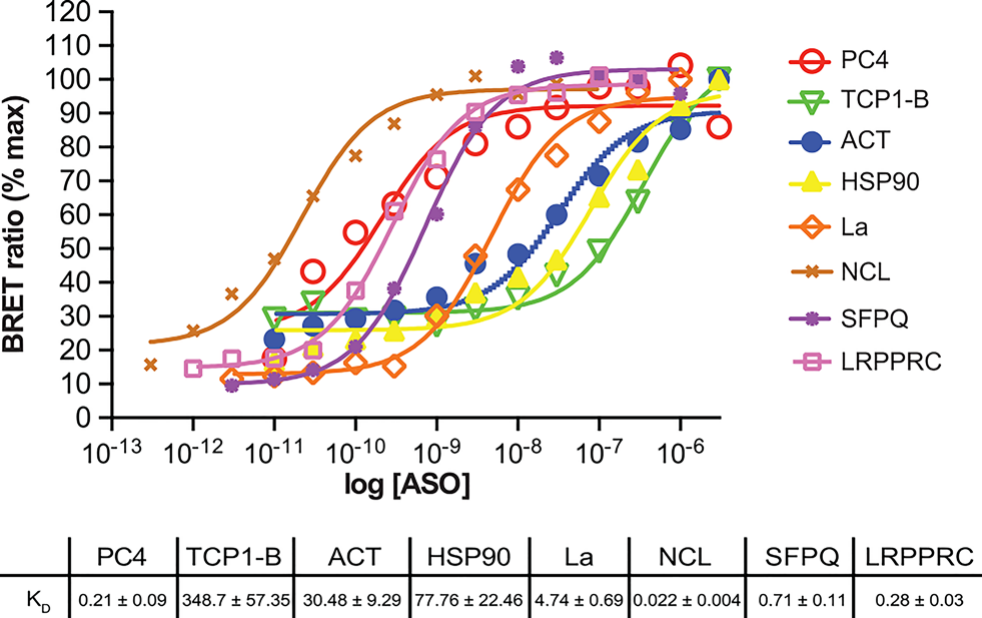
ASO/BRET binding affinities for various NLuc fusion proteins. NLuc protein fusions were constructed, expressed, and immunopurified as detailed in Materials and Methods. Binding affinities were determined by incubating 10^6^ RLU of immunopurified fusion protein with a 3’ ALEXA 594 conjugated 5-10-5 2’F gap-mer ASO (766638) at concentrations from 1 pM to 1 μM. Data are plotted as the percent of the maximal BRET ratio to control for differences in BRET amplitude for the various proteins. K_D_’s were determined using GraphPad Prism software. Similar experiments were performed for cEt (766636) and MOE (766634) gap-mer ASOs. The K_D_’s can be found in Table 1.

To further investigate the utility of BRET for ASO/protein affinity measurements, multiple NLuc fusion proteins were cloned then evaluated by direct binding with the same 2’F, MOE and cEt ASOs (S1 Table). The binding affinity for the 2’-F ASO was determined to be highly dependent on the conjugated protein, with K_D_s varying over 4 orders of magnitude (Fig 2). The affinity of the cEt and MOE ASOs also varied widely depending on the protein bound. A summary of affinity for all proteins can be found in Table 1. It is possible that a portion of this high variability in K_D_s may be due differences in sequence specificity of the proteins evaluated. However, it is interesting to note that the chemistry of the 2’ modification also had a variable effect on protein binding. For many proteins, affinity increased with increasing hydrophobicity of the 2’ modification (2’F>cEt>MOE), with almost 2 orders of magnitude difference in affinity between 2’ modifications for certain proteins. There also seems to be little correlation between the number or type of nucleic acid binding domains present in the protein and ASO affinity.

**Table 1:** Summary of NLuc fusion protein constructs and ASO/BRET binding affinities. Gene ID of full length protein is given. For certain proteins the full length protein was not cloned and the domains included in the NLuc fusion are indicated in parentheses. Known nucleic acid binding domains: RNA binding domain (RBD), DNA binding domain (DBD), Hybrid binding domain (HBD). K_D_’s (nM) for 5-10-5 2’F (766638), cEt (766636) and MOE (766634) gap-mer ASOs were determined using GraphPad Prism software.

Protein	NLuc	Domains	size (kD)	K_D_ Fl	K_D_ cEt	K_D_ MOE
LRPPRC	C	1-RBD	41.35	0.19	0.16	0.77
FUS	C	1-RBD	52.7	0.12	0.6	1.8
PC4	N	1-DBD	14.4	0.21	1.1	6.1
RPL5	N		20	0.6	1.3	3.7
NCL (RBD 1–4)	N/C	4-RBD	39	0.002	1.7	0.009
SFPQ	C	2-RBD	76	0.72	2.7	3.7
Ku70	C	2-DBD	69.9	3	4	15
RNAseH1	N/C	1-HBD	32	2	5	2
La	N/C	2-RBD	46.8	1.1	5.0	9.3
P54nrb	N/C	2-RBD	54	2.1	9.3	82.9
RPL11	N		34.4	9.8	17.4	15.7
HSP90 (mid)	C		47	98	43	167
Staufen	C	3-RBD	55		100	
TCP1-B	N		57	189	113	398
ACTB	N		42	28	295	252
NMP1	N/C	1-DBD	28.4	>1000	>1000	>1000
ANXA2	C	1-RBD	38	>1000	>1000	>1000

**Fig 3:**
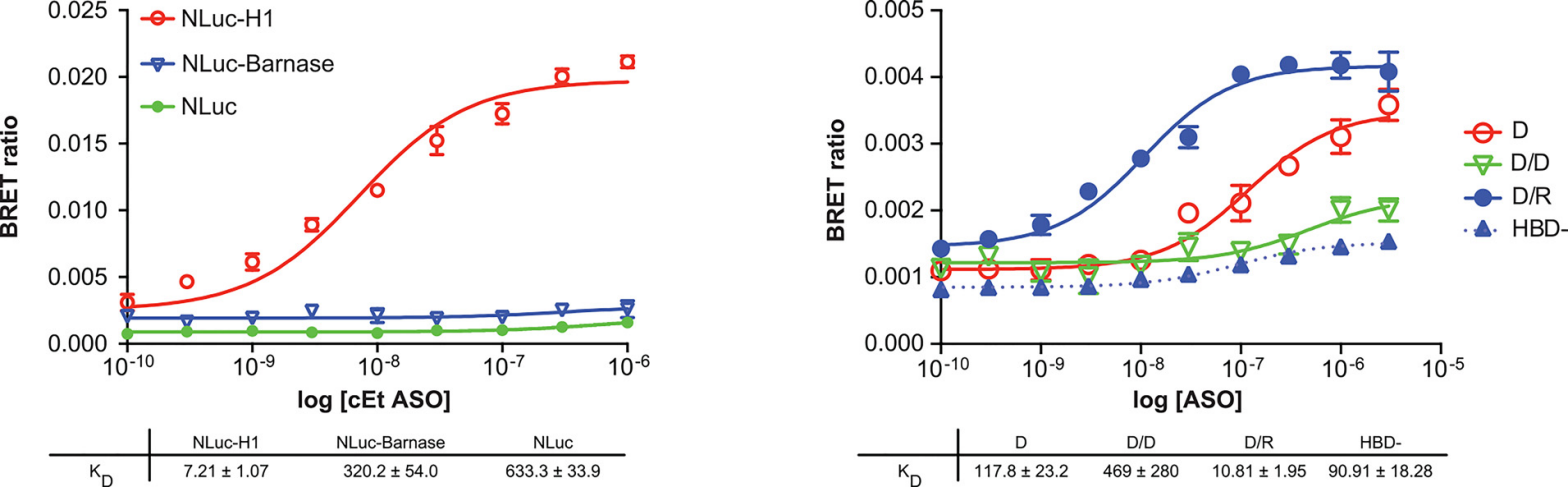
ASO binding to RNase H1 is highly specific. **A)** NLuc-RNAseH1 fusion (red) was constructed as described in Materials and Methods. The NLuc/Barnase fusion (blue) was produced from the plasmid pFN31K Nluc CMV-neo (Promega), whereas the NLuc only plasmid (green) was generated by deletion of the barnase coding region from the same plasmid. Proteins were expressed then immunopurified using and antibody specific for NLuc. Binding affinities were determined by incubating 10^6^ RLU of immunopurified protein with a 3’ ALEXA 594 conjugated 5-10-5 2’F (766638, solid lines) or MOE gap-mer (766634, dashed lines) ASOs at concentrations from 0.1 nM to 1 μM. **B)** The RNAse H1 hybrid binding domain (HBD) was deleted from NLuc/RNAseH1 by SDM. NLuc/RNAseH1 (solid lines) and NLuc/RNAseH1-HBD (dashed line) were expressed then immunopurified as above. Binding affinities were determined by incubating 10^6^ RLU of immunopurified protein with a 3’ ALEXA 594 conjugated PO ASO with or without complementary PO DNA or RNA. BRET rations were plotted and K_D_’s (nM) determined for single stranded DNA (D, red), DNA/DNA duplex (D/D, green), or DNA/RNA heteroduplex (D/R, blue). Data shown are mean ± SEM from 3 independent experiments.

To evaluate the specificity of the assay, the 3’-linked cEt gap-mer ASO was evaluated for binding to NLuc-RNAse H1. The same ASO was also evaluated for binding to NLuc alone, and NLuc-barnase (pFN31K Nluc), a protein not known to interact with ASOs. As has been previously observed [20], the PS ASO bound RNAse H1 with high affinity (Fig 3A, red line). In contrast, no BRET signal was observed for the NLuc-barnase (blue line) or Nluc (green line) control proteins. Similar results were observed with 2’-F or MOE PS gap-mer ASOs (data not shown). Together these data show that all of the ASO binding can be attributed to the fused protein and that NLuc does not contribute to binding.

In contrast to PS ASOs, PO ASOs have a much lower affinity for RNAse H1, unless they are part of DNA/RNA heteroduplex [20]. Furthermore, it has been demonstrated that the hybrid binding domain (HBD), located at the amino terminus of human RNase H1, mediates binding to the heteroduplex [21]. A 20-mer PO DNA linked with Alexa 594, was evaluated for binding to RNAse H1 in the BRET assay as single strand (D), or as a duplex with a complementary PO DNA (D/D) or PO RNA (D/R). Consistent with data obtained using purified RNAse H1 and a competitive cleavage assay [20], very little binding was observed to the DNA/DNA duplex (Fig 3B, green). While the ssDNA showed some affinity for RNAse H1 (red), the binding affinity of the DNA/RNA heteroduplex (blue) was more than 10 fold greater. Furthermore, deletion of the RNAse H1 HBD in the NLuc fusion, effectively ablated ASO binding (blue dashed lines).

**Fig 4:**
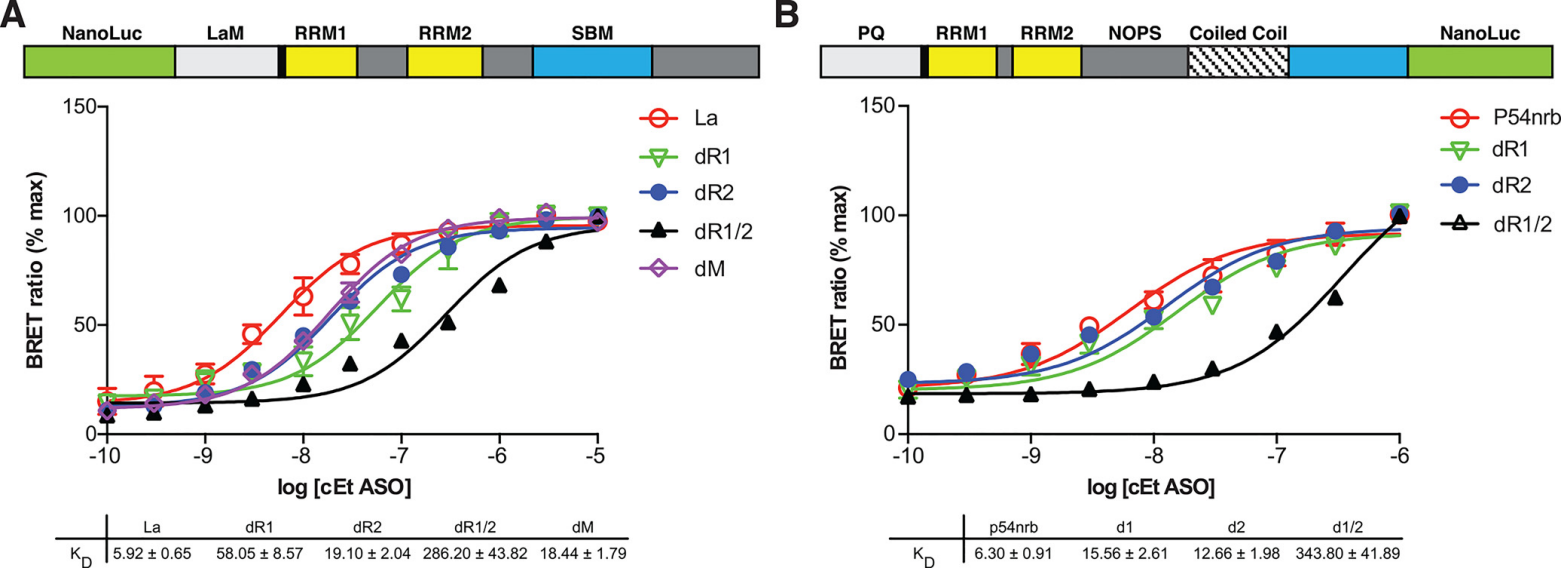
ASOs interaction with protein domains is specific and chemistry dependent. **A)** cEt gap-mer ASO binds to La RNA binding domains 1 and 2. Deletion mutants were generated from Nluc-La by SDM, then expressed and immunopurified using an antibody to La. Binding affinities were determined by incubating 10^6^ RLU of immunopurified protein with a 3’ ALEXA 594 conjugated 5-10-5 cEt gap-mer ASO (766636) at concentrations ranging from 100 pM to 10 μM. Data are plotted as the percent of the maximal BRET ratio to control for differences in BRET amplitude for the full length La (red), ∆La motif (violet), ∆RRM1 (green), ∆RRM2 (blue), or ∆RRM1/2 (black). **B)** cEt gap-mer ASO interacts with P54nrb at RRM1 and RRM2. Deletion mutants were generated from P54nrb/Nluc by SDM, then expressed and immunopurified using an antibody to P54nrb (Millipore). Binding affinities were determined by incubating 10^6^ RLU of immunopurified proteins with a 3’ ALEXA 594 conjugated 5-10-5 2’F gap-mer ASO (766638) at concentrations ranging from 100 pM to 1 μM. Concentration curves were plotted for BRET ratios using GraphPad PRISM software for full length P54nrb (red), ∆RRM1 (green), ∆RRM2 (blue), or ∆RRM1/2 (black). Data shown are mean ± SEM from 3–4 independent experiments.

To explore the domains involved in ASO- protein interactions, several deletion mutants were produced by site directed mutagenesis (SDM) for La protein. It is known that specific recognition of 3’ poly (U)-rich elements is mediated by the N-terminal domain (NTD) of La, which is comprised a La motif and an RNA recognition motif (RRM1) [18]. However, while it has also previously been shown that La also interacts with PS ASOs [6], little is known concerning the La domains which mediate interactions with ASOs. We deleted the La motif (dM), RRM1 (dR1), RRM2 (dR2), or both RRM1 and RRM2 (dR1/R2), then evaluated binding to the immunoprecipitated protein with the cEt ASO. An approximately 4 fold decrease in affinity was observed for the for the La motif (dM) or RRM2 (dR2) mutants as compared to the full length protein; while deletion of RRM1 (dR1) resulted in an almost 10 fold decrease in affinity and deletion of both RRM1 and RRM2 (dR1/R2) resulted in a ~50 fold decrease (Fig 4A). Similar results were observed for binding of 2’-F and MOE gap-mer ASOs to the same deletion mutants (data not shown). These data suggest that unlike the interaction of La with poly (U)-rich elements, PS ASOs can interact with either RRM1 or RRM2, with a slight preference for RRM1. Similar experiments were performed to identify the P54nrb domains which mediate ASO binding. Deletion of the P54nrb RRM1 and RRM2 domains resulted in a 2–3 fold decrease in affinity as compared to the full length protein (Fig 4B). However, deletion of both RRM1 and RRM2 strongly reduced ASO binding to P54nrb by more than 50 fold. These data suggest that PS ASOs do indeed interact with P54nrb via the RRM domains, however, in contrast to La, there is no strong preference for RRM1 over RRM2.

**Fig 5:**
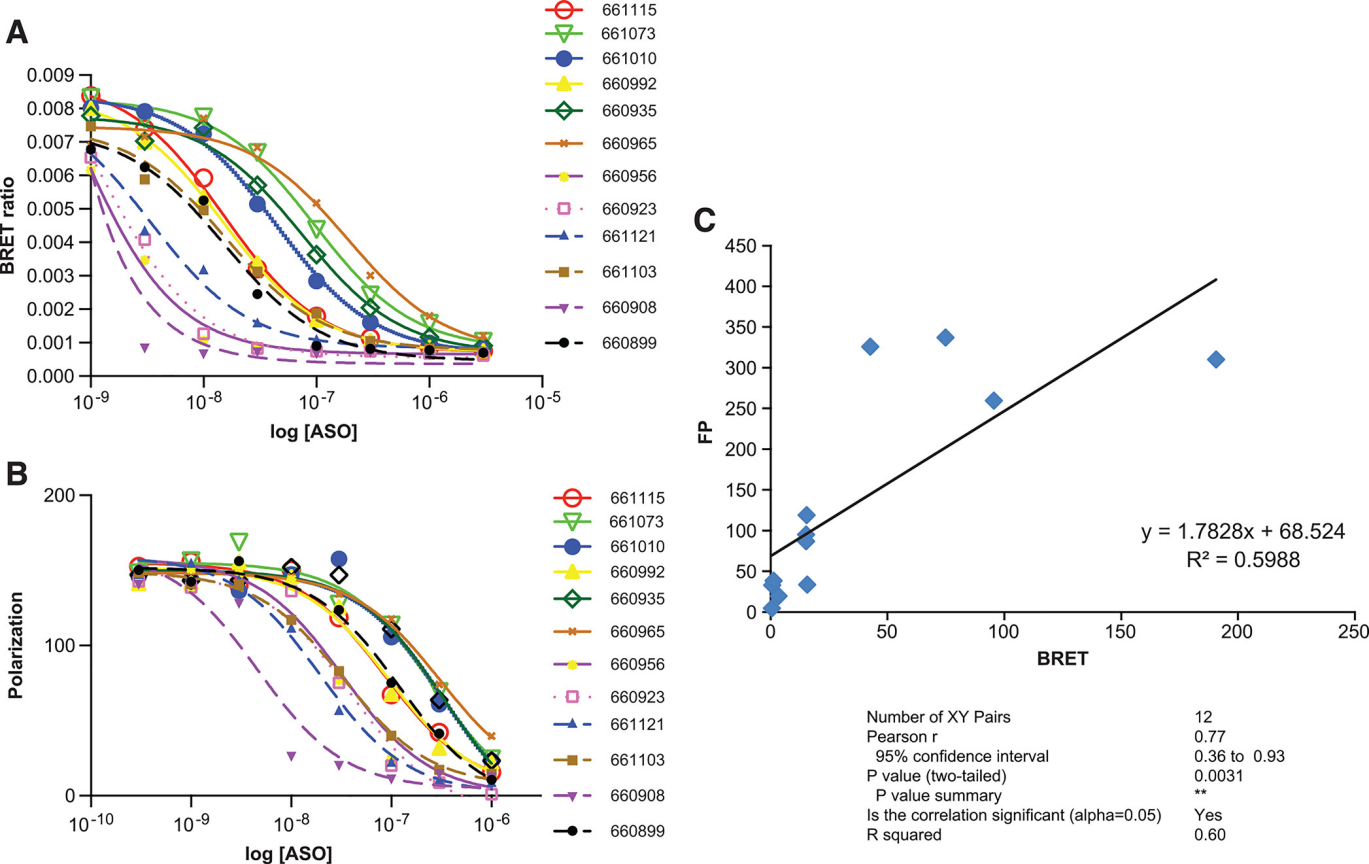
ASO binding affinity to P54nrb is highly sequence dependent. **A)** Competitive ASO/BRET binding of 3-10-3 cEt ASOs to P54nrb. Binding affinities were determined by incubating 10^6^ RLU of immunopurified NLuc/P54nrb fusion protein with 10 nM 3’ ALEXA 594 conjugated 5-10-5 2’F gap-mer ASO (766638) along with unconjugated TAAR5 3-10-3 cEt ASOs at concentrations ranging from 1 nM to 3 μM. Concentration curves are plotted for BRET ratios in the presence of each unconjugated ASO. TAAR5 ASO sequences and K_D_’s can be found in Table 2. **B)** P54nrb competitive fluorescent polarization assay. Competitive FP was performed with the same set of TAAR5 ASOs as detailed in Materials and Methods. C) Correlation between K_D_’s for ASO binding obtained by BRET and FP.

We next explored the effect of sequence on ASO affinity in the BRET assay. A series of 12, 3-10-3 cEt gap-mer ASOs (Table 2) targeting mouse trace amine associated receptor 5 (TAAR5), were evaluated for affinity to P54nrb by competitive binding in the BRET assay. ASO affinity to P54nrb was found to vary by over 2 orders of magnitude for this set of ASOs (Fig 5A). Binding of the same set of ASOs to purified P54nrb was also evaluated by fluorescence polarization (FP) (Fig 5B). While the variation in affinity between the tightest binding ASO and the weakest binding ASO was less for the FP assay than the BRET assay, the relative affinity was similar with a significant degree of correlation between the two methods (Fig 5C). Other proteins evaluated in the BRET assay with the same series of ASOs also demonstrated a great deal of variation in affinity, unique to each protein (S3 Fig). For P54nrb and RNAse H1, ASOs with the highest affinity all shared a GGG motif. No common sequence motifs were apparent for NCL or LRPPRC.

**Table 2:** TAAR5 ASO protein affinity. K_D_’s (nM) for competitive binding of ASOs to P54nrb obtained by BRET and FP.

IonisNo	Sequence	K_D_ (BRET)	K_D_ (FP)
660908	GGGAGGAGGACAGCTC	0.51 ± 0	4.601
660956	AACATGTCTGCCAGGG	0.71 ± 0.27	32.95
660923	CCAGCGGGTGGACTGT	1.22 ± 0.37	38.28
661121	CCCTCCCTCCCGCTAG	3.39 ± 0.84	19.53
660992	ACCCTGCCACGATGTA	15.19 ± 0.73	95.06
661115	TCAGTCATGGTATAAA	15.22 ± 1.49	86.93
660899	CTGGGAACTGGTCACC	15.36 ± 3.63	118.8
661103	TGAGAAGATCTCCCGG	15.67 ± 3.01	33.57
661010	CTTCTAGCCACTGGCT	42.69 ± 4.17	325.6
660935	CCACTGCGCAGGCCAG	74.83 ± 13.02	336.9
661073	GTTAAGAAGGCTGTCC	95.54 ± 12.16	259.7
660965	TCCACAGAGCGGACTG	190.6 ± 47.12	309.9

**Fig 6:**
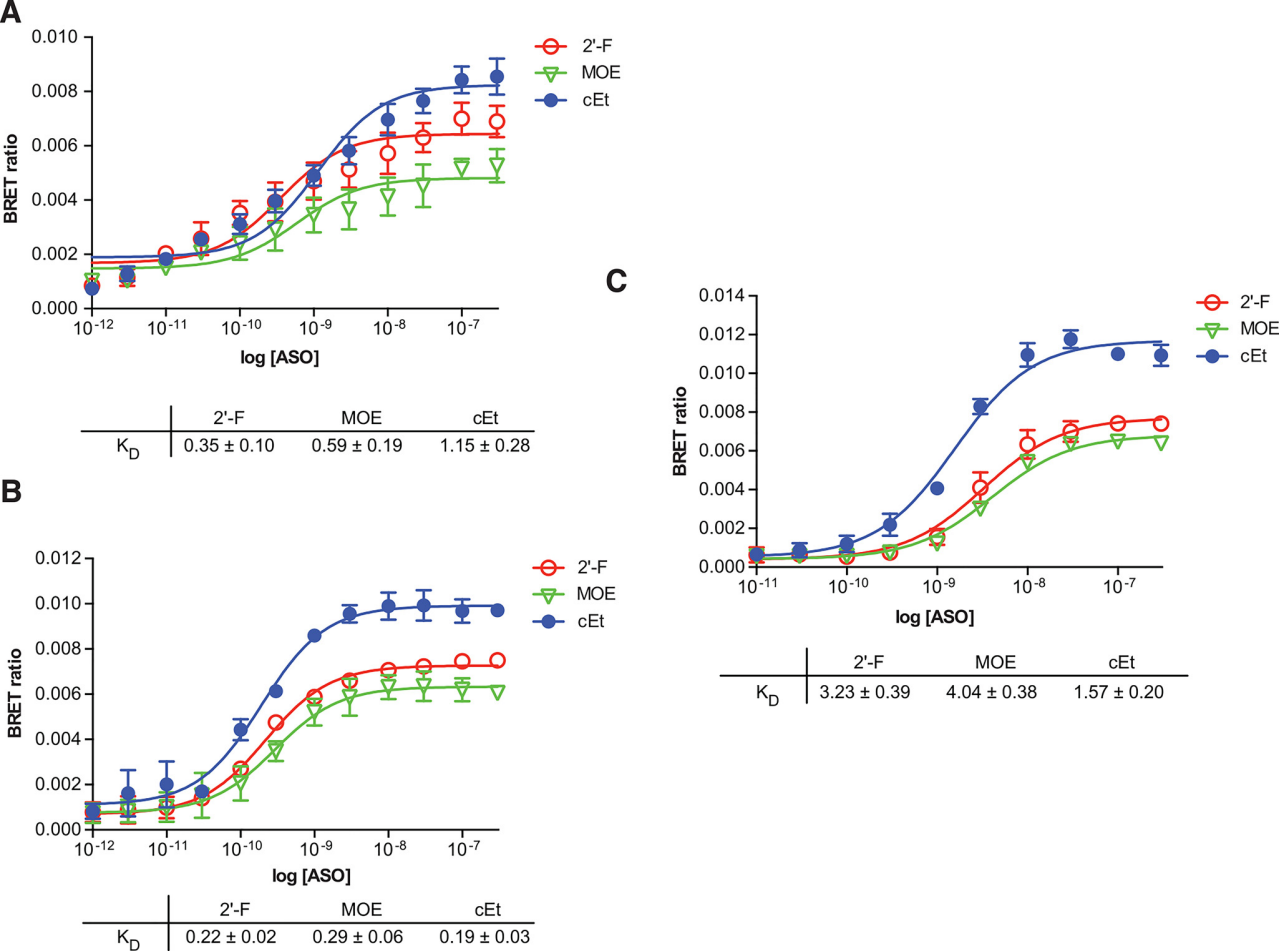
ASO/BRET in lysates and permeabilized cells. LRPPRC/NLuc was constructed as detailed in Materials and Methods. The fusion protein was expressed by transient transfection in Hela cells. Cell lysate was prepared and the fusion protein immunopreciptated from ½ of the lysate with an antibody to NLuc. For ASO/BRET using the IP’ed protein **(A)** and cell lysate **(B)**, binding affinities were determined by incubating 10^6^ RLU of LRPPRC/NLuc fusion protein with 3’ ALEXA 594 conjugated 5-10-5 gap-mer ASO at concentrations between 3 pM to 300 nM. Concentration curves were plotted for BRET ratios using GraphPad PRISM software for 2’F (766638, red), MOE (766634, green), or cEt (766636 blue) gap-mer ASOs. For Nano BRET in intact cells **(C)**, the HeLa cells expressing the LRPPRC/NLuc fusion were seeded in 96-well plates at 5000 cells/well. 24 hours after the initiation of transfection, cells were permeabilzed with 25 ng/mL digitonin in OptiMEM media plus 5-10-5 gap-mer ASO at concentrations between 3 pM to 300 nM. After a 15 minute incubation NanoGlo substrate was added and BRET ratios determined as above. Data shown are mean ± SEM from 3 independent experiments.

In an attempt to extend the utility of the assay, BRET was performed with crude lysates or in intact cells as detailed in Materials and Methods. The 2’F, MOE, or cEt ASO bound the immunopurified LRPPRC fusion protein with K_D_’s of 0.35, 0.59 and 1.15 nM respectively (Fig 6A). Affinities for the 2’F and MOE ASO in the crude lysate were similar (K_D_’s of 0.22 and 0.29 nM), whereas the K_D_ for the cET was slightly lower (0.19 nM) than observed with the purified fusion protein (Fig 6B). Live cells were permeabilized with digitonin (DIG) in the presence of ALEXA-conjugated ASO, followed by addition of NLuc substrate. In these intact, permeablized cells, the BRET affinity was reduced by approximately 10–15 fold relative to the lysate or IP-ed fusion protein (Fig 6C). For other proteins, the difference in ASO affinity between intact cells and immunopurified protein was even more significant (S4 Fig). In general, binding was found to be more significantly reduced in lysates and DIG permeabilized cells for those proteins with lower ASO affinity. This may be due to competition with higher affinity and/or more abundant proteins in the cells and lysate relative to the immunopurified protein, although the actual concentration of the ASO in the permeabilzed cell may be lower than that added to the media. It should also be noted that in the absence of efficient permeabilizing agents (S4C Fig), ASO binding was not detectable. Delivery of ASO to the cells by cationic lipid also did not result in significant BRET signal (data not shown).

Staufen1 (STAU1)-mediated mRNA decay (SMD) is an mRNA degradation process in mammalian cells that is mediated by the binding of STAU1 to a STAU1-binding site (SBS) within the 3'-untranslated region (3'-UTR) of target mRNAs [22]. STAU1 is a member of the family of double-stranded RNA (dsRNA)-binding proteins involved in the transport and/or localization of mRNAs to different subcellular compartments and/or organelles. These proteins are characterized by the presence of multiple dsRNA-binding domains which are required to bind RNAs having double-stranded secondary structures [23]. An amino-terminal NLuc/STAU1 fusion was evaluated for binding specificity to dsRNA in the BRET assay. A 20 nucleotide, 3’-Alexa-linked RNA was hybridized to a complementary 40-mer RNA (RNA/RNA) or 40-mer DNA of the same sequence (RNA/DNA) (S1 Table). Binding of the RNA/RNA or RNA/DNA duplex, as well as the 3’-Alexa-linked ssRNA and ssDNA 20-mers, was then evaluated by BRET. The affinity of the ds RNA duplex was determined to be approximately 3 nM (red line), in close agreement with the K_D_ previously obtained using purified protein and a filter binding assay [24]. In contrast, STAU1 affinity for ssRNA (blue), ssDNA (black), or RNA/DNA heteroduplex (green) was reduced by ~30–50 fold relative to the affinity for dsRNA, with K_D_’s ranging from 130–207 nM.

**Fig 7:**
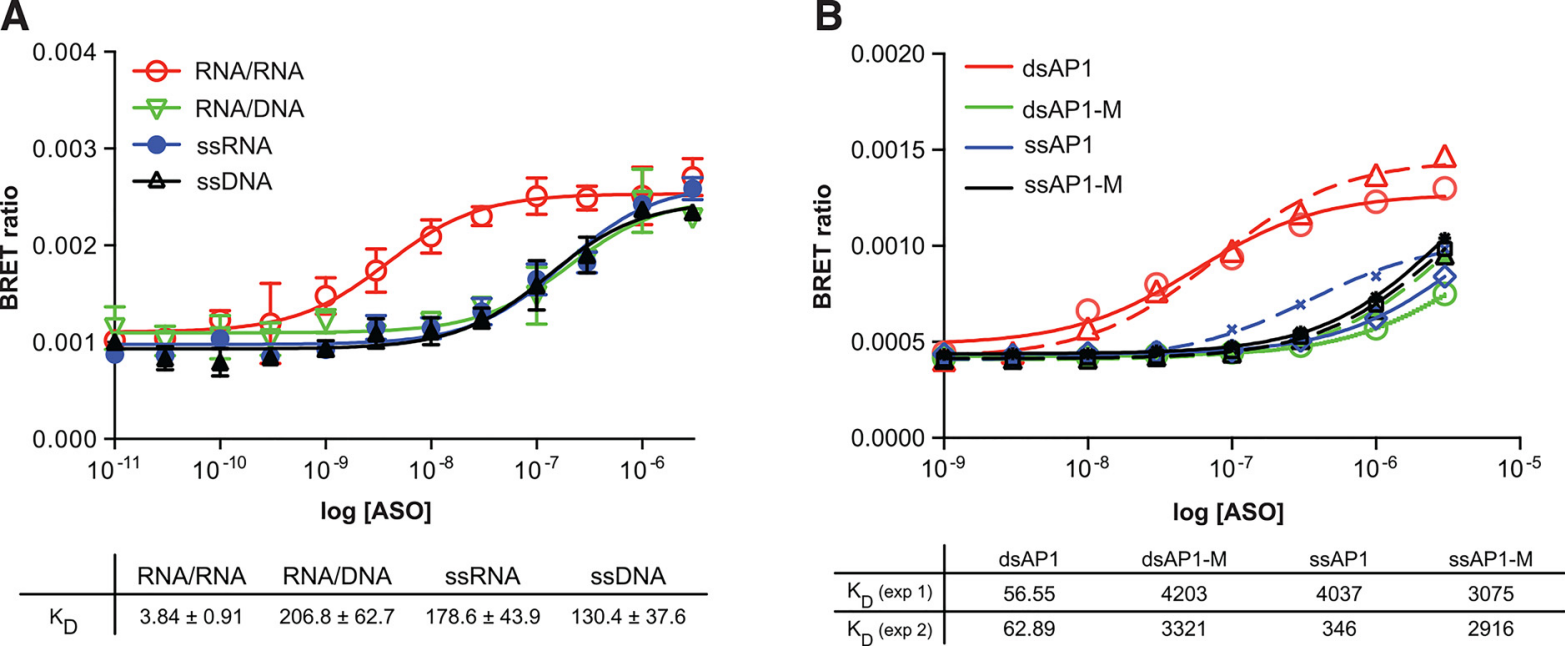
BRET with RNA and DNA binding proteins. **A)** Staufen 1 specifically binds RNA duplexes. An NLuc/STAU1 fusion was constructed and expressed as detailed in Materials and Methods. The protein was immunoprecipitated from the cell lysate using an STAU1 antibody (Millipore AB5781), then binding affinities were determined by incubating 10^6^ RLU of immunopurified fusion protein with a 3’ ALEXA 594 conjugated PO RNA 20-mer hybridized with a 40 nt complementary RNA strand (RNA/RNA; red) or a 3’ ALEXA 594 conjugated PO DNA 20-mer hybridized with the 40 nt complementary RNA strand (RNA/DNA; green) at concentrations ranging from 10 pM to 3 μM. Binding of the ssRNA (blue) and ssDNA (black) was also evaluated. Data is plotted as BRET ratio vs concentration RNA and represent mean ± SEM from 3 independent experiments. **B)** C-Jun binding to AP1 dsDNA by BRET. An amino-terminal NLuc fusion was constructed with the C-Jun protein (NLuc/cJun). The fusion protein was expressed by transient transfection in Hela cells which were subsequently seeded in 96 well plates at 5000 cells/well. 24 hours after the initiation of transfection, cells were permeabilzed with 25 ng/mL digitonin in OPtiMEM media plus PO DNA comprising the ds AP1 consensus sequence or a mutant AP1 site (AP1-M) at concentrations between 1 nM to 10 μM. Single stranded DNAs without the complementary DNA strand were also included in the BRET assay. After a 15 minute incubation NLuc substrate was added and BRET ratios determined as detailed above. Concentration response curves and K_D_’s (nM) for two independent experiments are shown.

The interaction of proteins with DNA is central to the control of many cellular processes including DNA replication, recombination and repair, transcription, and viral assembly. One important technique for studying gene regulation and determining protein:DNA interactions is the electrophoretic mobility shift assay (EMSA) [25]. One advantage of EMSA over other binding assays is that the source of the DNA-binding protein may be a crude nuclear or whole cell extract. Furthermore, EMSA can be used qualitatively to identify sequence-specific DNA-binding proteins (such as transcription factors) in crude lysates and, in conjunction with mutagenesis, to identify the important binding sequences within a given gene’s upstream regulatory region. However, EMSA is relatively low throughput. The AP-1 transcription factor is composed of a mixture of homo- and hetero-dimers formed between Jun and Fos proteins [26]. An amino-terminal NLuc fusion was constructed with the c-Jun protein. We then evaluated binding to an Alexa-conjugated AP1 double stranded DNA consensus binding site in DIG permeabilized cells. Binding to the dsAP1 site was highly specific, with K_D_’s between 56.6 and 62.9 nM (Fig 7B, red lines). Binding in the BRET assay to AP1 was highly specific, with the affinity for AP1 mutated at the consensus binding site (green lines) or single stranded DNA (blue, black) approximately 2 orders of magnitude less than the ds consensus site.

## Discussion

In this manuscript we present a novel rapid throughput BRET assay designed to measure nucleic acid/protein interactions. The assay takes advantage of the high intensity luminescence generated with NLuc fusion proteins, which is transferred to an acceptor fluorophore conjugated to an oligonucleotide binding partner. This assay can generate K_D_’s and relative K_D_’s for interactions between ASOs and proteins (Fig 1). In addition, information can be generated which provides an estimate of the relative distance between the binding site and the end of the protein. Furthermore, in our experience, the assay is highly reproducible. For example, K_D_ curves for the binding of a 3’ ALEXA 594 conjugated 5-10-5 ASO cEt gap-mer ASO generated using 3 independent preparations of immunopurified p54nrb-Nluc fusion protein, yielded highly similar results with little standard error (S5 Fig). The standard error was typically low for independent replicates in other experiments as well (Figs 1, 3, 4, 6 and 7).

A FRET-based assay for the analysis of protein nucleic acids was recently described which relies on genetic incorporation of a fluorescent amino acid into the protein of interest [27]. There are several advantages of the ASO/BRET assay over this and other current methods [28] used to detect and quantify nucleic acid/protein interactions. First, an extensive purification of the protein of interest is not required. This eliminates the need for protein denaturation/renaturation and supports the evaluation of native ASO/protein interactions using immunoprecipitates. Further, for many proteins, crude cell lysates from cells transiently transfected with an NLuc fusion protein can even be used, eliminating the need for the development of specific antibodies to the protein of interest. Since extensive protein purification is not required, the assay also supports evaluation of ASO interactions with protein complexes rather than a single purified protein. In addition, mutant proteins can be rapidly generated by SDM, then quickly expressed and evaluated (Fig 4). It should be noted that as the size of the fused protein increased, the amplitude of the BRET signal was reduced, although this is not always the case (S2 Fig, Table 1). This is likely due to the increased distance between the bound ASO fluorophore and the amino- or carboxy-terminal fused NLuc. The decreased BRET amplitude results in less accurate BRET ratios, and suggests that there is a maximum protein size of which is practical for the assay, but which may also depend on the relative location of the ASO binding site on the protein.

The ASO/BRET assay is highly specific and quantitative. We detected no ASO binding to Nluc alone or to NLuc fused to Barnase, a protein not known to interact with nucleic acids (Fig 3A). In addition, the large difference in ASO affinity for known nucleic acid binding proteins (Fig 2, S2 Fig, Table 1), indicates that the K_D_’s generated in the assay are accurate and specific, and that the assay has a linear dynamic range extending over several orders of magnitude. It is interesting to note that there was little correlation between the number of nucleic acid binding domains present in a given protein and overall affinity for ASOs (Table 1). Finally, PO ASO interactions with NLuc/RNAseH1 (Fig 3B) are consistent with previously published data showing a preference for binding of an RNA/DNA heteroduplex at the RNAse HI HBD [20,21].

We observed large differences in ASO affinity and in the site of interaction of the ASO on the protein based upon the chemistry of the 2’ modification. In general, ASOs with the more hydrophobic 2’ modified bases (2’F>cEt>MOE) bound with higher affinity (Table 1, Fig 2, S2 Fig), however this was not always the case as with RNAse H1 and Nucleolin. The interaction of La protein with 3' poly (U)-rich elements is known to occur primarily through the LaM domain and RRM1 [18], however, the PS ASOs we evaluated also appear to interact with the RRM2 domain as well as LaM and RRM1. (Fig 4A). Similarly, PS ASOs interact with P54nrb at both RRM1 and RRM2 (Fig 4B). Clearly, further studies need to be undertaken to understand the nature and specificity of these interactions; however these results emphasize the utility of this assay.

It was also determined that the sequence of an ASO can have profound effects on affinity. For example, affinity of 12 cEt ASOs to P54nrb varied by over 2 orders of magnitude (Fig 5A). Similar sequence dependent differences in affinity were observed for other proteins (S3 Fig), however no clear sequence motifs emerged in the high affinity ASOs and there was little correlation between antisense activity and ASO affinity for any protein (Table 2). Clearly many more sequences must be evaluated in order for any rules to be determined or correlations to be observed.

For certain high affinity proteins such as LRPPRC and NCL (Fig 6, S4B Fig) ASO/BRET can be performed with crude lysates or even in intact cells. However, for lower affinity proteins such as RpL5 and P54nrb, a large difference was observed in K_D_’s using the immunopurified fusion protein relative to performing the assay in intact cells (S4A and S4C Fig). It is unlikely that this difference is due to inefficient cell permeabilization or quenching by the media, since affinities obtained from permeabilized, intact cells and cell lysates were similar, especially for RPL5. More likely, reduced affinities may be the result of interaction of the ASO in the cell with more abundant or higher affinity proteins present in the intact cell or lysate which compete for ASO binding with lower affinity NLuc fusion proteins. It should also be noted that in the absence of efficient permeabilizing agents, ASO binding was not detectable (S4C Fig). Delivery of ASO to the cells by cationic lipid also did not result in significant BRET signal (data not shown). This may be the result of differences in cellular compartmentalization between the transfected ASO and fusion protein, or more likely, transfection does not deliver ASO in high enough concentrations to produce a detectable BRET signal.

To expand the utility of the assay beyond ASO/protein interactions, we evaluated interactions of known DNA and RNA binding proteins with oligonucleotides by BRET. Stau1 has been demonstrated to bind double-stranded RNAs [23]. In the BRET assay, STAU1 was shown to bind preferentially to dsRNA over ssRNA or an RNA/DNA heteroduplex (Fig 7A). The affinities obtained by BRET were in close accord with those previously obtained using purified protein and a filter binding assay [24]. BRET was also demonstrated to be a high throughput alternative for transcription factor binding by EMSA. Binding of the NLuc/c-Jun fusion protein was shown to be highly specific to the dsAP1 site, with a K_D_ of approximately 80 nM with virtually no binding to a mutant dsAP1 site or the single stranded DNA (Fig 7B). While this number is somewhat higher than that previously published for binding of AP1 by Fos/Jun heterodimer by EMSA (4-12nM) [29,30], it may be attributable to the much less complex binding environment in the EMSA as compared to the cellular BRET assay.

Finally, the high intensity of ASO/BRET raises the potential for quantifying energy transfer between ASOs and specific proteins in individual cells by microscopic imaging. Such experiments would provide insight into ASO uptake, trafficking, and distribution, leading to improvements in specificity and potency of antisense therapeutics.

## Supporting Information

S1 Fig Western blot of representative NLuc fusion proteins. 6 x 10^5^ HeLa cells were transfected with NLuc fusion constructs using Effectene transfection reagent. After 24 hours cells were collected and lysates prepared for Western blot analysis as detailed in Materials and Methods. **A)** Anti NLuc pAb (Promega). **B)** Blot in panel A was stripped then re-probed with gene specific antibodies for P54nrb, Millipore 05–950; SFPQ, Abcam Ab38148); NPM1, Abcam Ab10530; C-Jun, Abcam Ab31419; Actin-B, Abcam Ab20272
(PDF)

S2 Fig ASO/BRET affinity for La protein. **A)** La fusion proteins were immunopurified as detailed in Materials and Methods and subsequently incubated with Alexa 594 conjugated 5-10-5 cEt gap-mer ASO at concentrations ranging from 10 pM to 10 μM. BRET ratios were determined for La-NLuc (green, black) or NLuc-La (red, blue), with either a 3’ conjugated (766636) or 5’ conjugated (766635) ASO. Concentration response curves and K_D_’s (nM) for two independent experiments are shown. **B)** ASO/BRET binding affinity varies with chemistry of the 2’ modification. ASO/BRET assay was performed with NLuc-La fusion and 5’ conjugated 5-10-5 ASOs at concentrations ranging from 10 pM to 10 μM. 2’F, red; cEt, blue; MOE, green. **C)** Relative affinities for 2’F (red), cET (blue), and MOE (green) gap-mer ASO as determined by competitive ASO binding to the NLuc-La fusion protein in the BRET assay. 10 nM 3’ Alexa conjugated cEt ASO (766636) was competed with unconjugated 5–10–5 2′-F (red), MOE (green), or cEt (blue) gap-mer ASO at concentrations from 0.1 to 1000 nM. Relative K_D_’s are shown. Data in panels B and C are mean ± SEM from 3–4 independent experiments.
(PDF)

S3 Fig Sequence dependent binding of 3-10-3 cEt gap-mer ASOs. NLuc protein fusions were constructed for RNAse H1 **(A)**, Nucleolin **(B)**, and LRPPRC **(C)**, then expressed, and immunopurified as detailed in Materials and Methods. Competitive binding affinities were determined by incubating 10^6^ RLU of immunopurified NLuc fusion protein with 10 nM 3’ ALEXA 594 conjugated 5-10-5 cEt gap-mer ASO along with unconjugated TAAR5 3-10-3 cEt ASOs at concentrations ranging from 3 pM to 3 μM. Concentration curves are plotted for BRET ratios in the presence of each unconjugated ASO. TAAR5 ASO sequences and K_D_’s can be found in S4 Table.
(PDF)

S4 Fig ASO NanoBRET in permeabilized cells. P54nrb-NLuc (A), NLuc-NCL **(B)**, and NLuc/RPL5 **(C)** fusion proteins were expressed by transient transfection in Hela cells. Cell lysates were prepared and the fusion protein immunopreciptated with an antibody to NLuc. For ASO NanoBRET using the IP’ed protein (red) or crude lysates (green), binding affinities were determined by incubating 10^6^ RLU of fusion protein with a 3’ ALEXA 594 conjugated 5-10-5 cEt gap-mer ASO at the indicated concentrations. Concentration curves were plotted for BRET ratios using GraphPad PRISM software. For Nano BRET in intact cells, the HeLa cells expressing the fusion protein were seeded in 96-well plates at 5000 cells/well. 24 hours after the initiation of transfection, cells were permeabilzed with 25–50 ng/mL digitonin in OptiMEM (blue) or PBS (blue dashed) plus the cEt gap-mer ASO at concentrations between 1 nM and 1 uM. After a 30 minute incubation NLuc substrate was added and BRET ratios determined as above. For thr RPL5 fusion, cells were also treated in OptiMEM in the presence of 0.2% saponin (black) or in OptiMEM with no permeabilizing agent (brown).
(PDF)

S5 Fig ASO NanoBRET reproducibility. **A)** P54nrb/Nluc fusion protein was expressed by transient transfection of 3 different plates of Hela cells. Cell lysates were prepared and the fusion proteins immunoprecipitated with an antibody to P54nrb. Binding affinities were determined in separate experiments by incubating 10^6^ RLU of fusion protein with a 3’ ALEXA 594 conjugated cEt gap-mer ASO at concentrations ranging from 10 pM to 3 uM. Binding curves are shown for each of the biological replicates and as well as the mean ± SEM for the 3 samples (black).
(PDF)

S1 Table ASOs used in study. For gap-mer ASOs, 2’ modified bases (2’-alpha-flouro, (S)-cEt, and MOE) are indicated by bold type. The Ionis numbers of the gap-mer ASO are in parentheses. For AP1 oligonucleotides, the AP1 consensus site is underlined and consensus binding site mutations are indicated in red.
(PDF)

S2 Table Sequences of PCR primers used to generate cDNAs for directional in-frame cloning with NLuc. (PDF)

S3 Table Sequences of SDM primers used to generate deletion mutants for p54nrb, La, and RNAse H1. (PDF)

S4 Table TAAR5 ASOs activity and protein affinity. 20,000 MEF cells were treated with 3.5 uM ASO by electroporation in 96 well pltes in a total volume of 100 ul. 24 hours later total RNA was purified and levels of TAAR5 mRNA accessed by qRT/PCR as described previously [11]. qRT/PCR FP: TGCTACCAGGTGAATGGGTCTT, RP: TGCGCAGGCCAGATAGATG, probe: AGGACAGTCCACCCGCTGGCC. For each ASO data is presented as the percent of mock treated control for 3 replicates. K_D_’s (nM) for NanoBRET ASO binding were determind us GraphPad PRISM software.
(PDF)
