# Supplementary material for: Development of a Quantitative BRET Affinity Assay for Nucleic Acid-Protein Interactions
Source: PLoS One. 2016 Aug 29;11(8):e0161930. doi: 10.1371/journal.pone.0161930 (PMC5003356; doi:10.1371/journal.pone.0161930)
Supplement: S1 Fig — 6 x 10^5^ HeLa cells were transfected with NLuc fusion constructs using Effectene transfection reagent. After 24 hours cells were collected and lysates prepared for Western blot analysis as detailed in Materials and Methods. A) Anti NLuc pAb (Promega). B) Blot in panel A was stripped then re-probed with gene specific antibodies for P54nrb, Millipore 05–950; SFPQ, Abcam Ab38148); NPM1, Abcam Ab10530; C-Jun, Abcam Ab31419; Actin-B, Abcam Ab20272 (PDF) [file pone.0161930.s001.pdf]

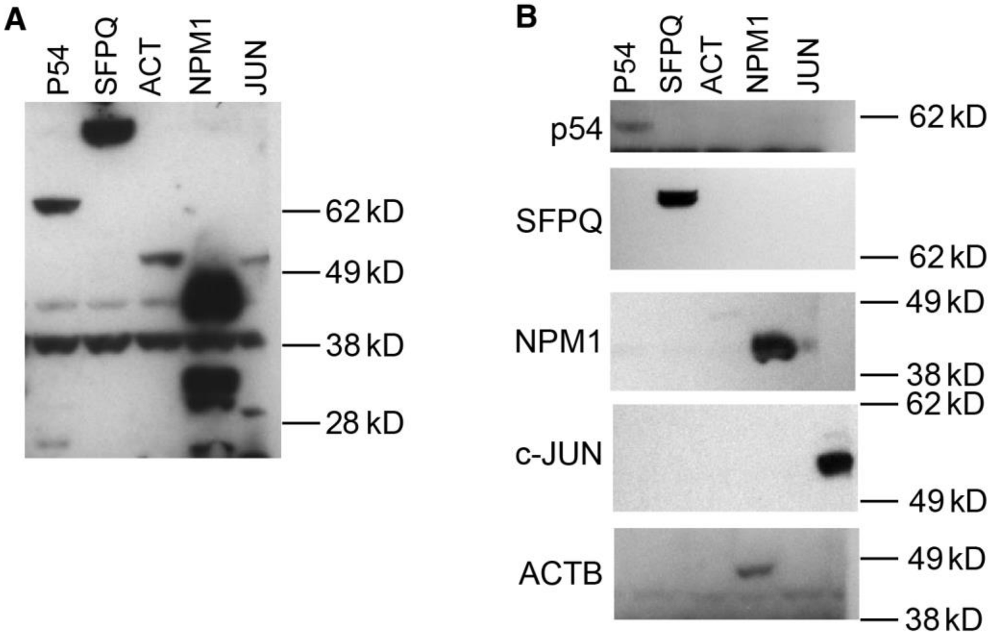

S1 Fig. Western blot of representative NLuc fusion proteins. 6 x 10^5 HeLa cells were transfected with NLuc fusion constructs using Effectene transfection reagent. After 24 hours cells were collected and lysates prepared for Western blot analysis as detailed in Materials and Methods. **A)** Anti NLuc pAb (Promega). **B)** Blot in panel A was stripped then re-probed with gene specific antibodies for P54nrb, Millipore 05-950; SFPQ, Abcam Ab38148); NPM1, Abcam Ab10530; C-Jun, Abcam Ab31419; Actin-B, Abcam Ab20272.
