# Supplementary material for: Development of a Quantitative BRET Affinity Assay for Nucleic Acid-Protein Interactions
Source: PLoS One. 2016 Aug 29;11(8):e0161930. doi: 10.1371/journal.pone.0161930 (PMC5003356; doi:10.1371/journal.pone.0161930)
Supplement: S2 Fig — A) La fusion proteins were immunopurified as detailed in Materials and Methods and subsequently incubated with Alexa 594 conjugated 5-10-5 cEt gap-mer ASO at concentrations ranging from 10 pM to 10 μM. BRET ratios were determined for La-NLuc (green, black) or NLuc-La (red, blue), with either a 3’ conjugated (766636) or 5’ conjugated (766635) ASO. Concentration response curves and KD’s (nM) for two independent experiments are shown. B) ASO/BRET binding affinity varies with chemistry of the 2’ modification. ASO/BRET assay was performed with NLuc-La fusion and 5’ conjugated 5-10-5 ASOs at concentrations ranging from 10 pM to 10 μM. 2’F, red; cEt, blue; MOE, green. C) Relative affinities for 2’F (red), cET (blue), and MOE (green) gap-mer ASO as determined by competitive ASO binding to the NLuc-La fusion protein in the BRET assay. 10 nM 3’ Alexa conjugated cEt ASO (766636) was competed with unconjugated 5–10–5 2′-F (red), MOE (green), or cEt (blue) gap-mer ASO at concentrations from 0.1 to 1000 nM. Relative KD’s are shown. Data in panels B and C are mean ± SEM from 3–4 independent experiments. (PDF) [file pone.0161930.s002.pdf]

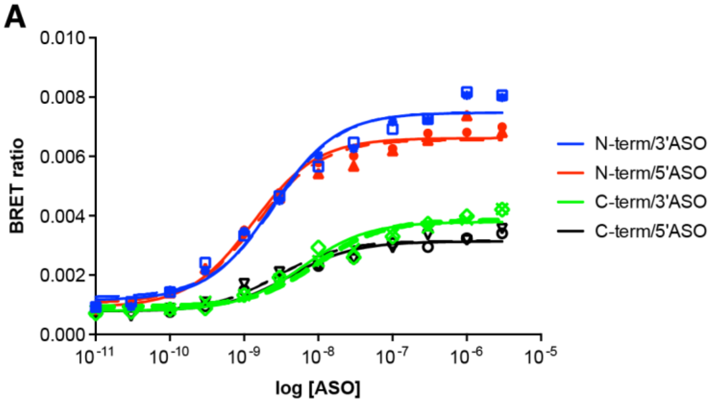

	C-term/5'ASO	N-term/5'ASO	C-term/3'ASO	N-term/3'ASO
K_D (exp 1)	4.10	1.31	6.24	2.4
K_D (exp 2)	2.79	1.52	9.67	2.61

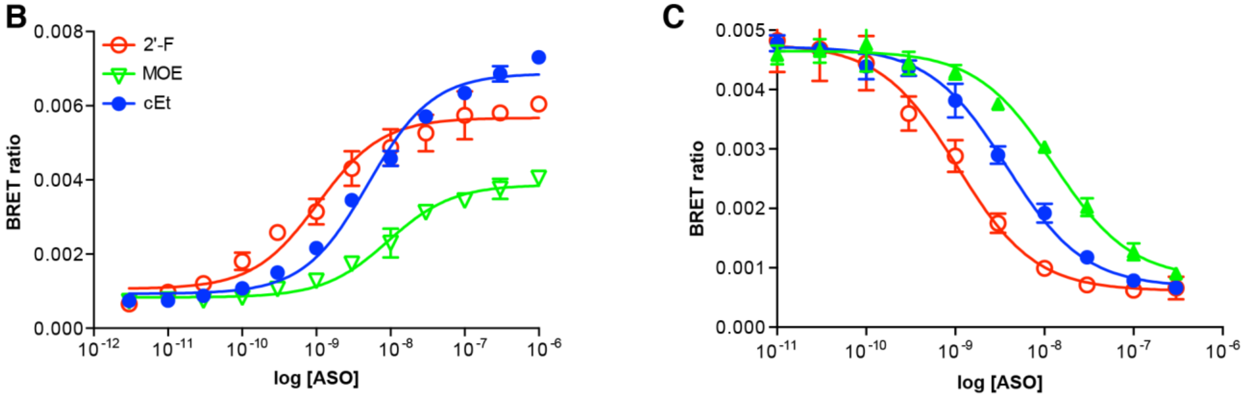

	2'-F	MOE	cEt
K_D	1.05 ± 0.17	9.29 ± 1.13	5.01 ± 0.52

	2'-F	MOE	cEt
K_D	1.06 ± 0.15	12.71 ± 2.01	3.78 ± 0.46

S2 Fig. ASO/BRET affinity for La protein. **A)** La fusion proteins were immunopurified as detailed in Materials and Methods and subsequently incubated with Alexa 594 conjugated 5-10-5 cEt gap-mer ASO at concentrations ranging from 10 pM to 10 µM. BRET ratios were determined for La-NLuc (green, black) or NLuc-La (red, blue), with either a 3’ conjugated (766636) or 5’ conjugated (766635) ASO. Concentration response curves and K_D’s (nM) for two independent experiments are shown. **B)** ASO/BRET binding affinity varies with chemistry of the 2’ modification. ASO/BRET assay was performed with NLuc-La fusion and 5’ conjugated 5-10-5 ASOs at concentrations ranging from 10 pM to 10 µM. 2’F, red; cEt, blue; MOE, green. **C)** Relative affinities for 2’F (red), cET (blue), and MOE (green) gap-mer ASO as determined by competitive ASO binding to the NLuc-La fusion protein in the BRET assay. 10 nM 3’ Alexa conjugated cEt ASO (766636) was competed with unconjugated 5–10–5 2′-F (red), MOE (green), or cEt (blue) gap-mer ASO at concentrations from 0.1 to 1000 nM. Relative K_D’s are shown. Data in panels B and C are mean ± SEM from 3-4 independent experiments.
