# Supplementary material for: Development of a Quantitative BRET Affinity Assay for Nucleic Acid-Protein Interactions
Source: PLoS One. 2016 Aug 29;11(8):e0161930. doi: 10.1371/journal.pone.0161930 (PMC5003356; doi:10.1371/journal.pone.0161930)
Supplement: S3 Fig — NLuc protein fusions were constructed for RNAse H1 (A), Nucleolin (B), and LRPPRC (C), then expressed, and immunopurified as detailed in Materials and Methods. Competitive binding affinities were determined by incubating 106 RLU of immunopurified NLuc fusion protein with 10 nM 3’ ALEXA 594 conjugated 5-10-5 cEt gap-mer ASO along with unconjugated TAAR5 3-10-3 cEt ASOs at concentrations ranging from 3 pM to 3 μM. Concentration curves are plotted for BRET ratios in the presence of each unconjugated ASO. TAAR5 ASO sequences and KD’s can be found in S4 Table. (PDF) [file pone.0161930.s003.pdf]

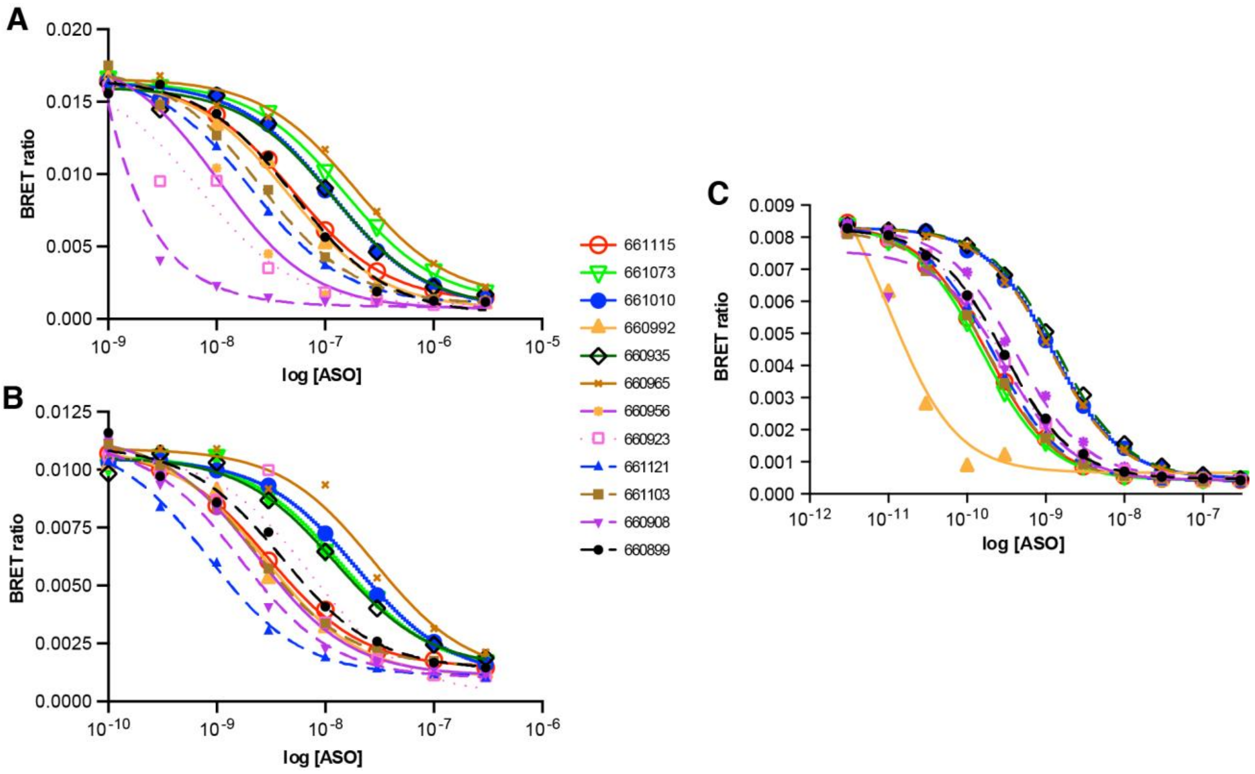

S3 Fig. Sequence dependent binding of 3-10-3 cEt gap-mer ASOs. NLuc protein fusions were constructed for RNAse H1 **(A)**, Nucleolin **(B)**, and LRPPRC **(C)**, then expressed, and immunopurified as detailed in Materials and Methods. Competitive binding affinities were determined by incubating 10^6 RLU of immunopurified NLuc fusion protein with 10 nM 3' ALEXA 594 conjugated 5-10-5 cEt gap-mer ASO along with unconjugated TAAR5 3-10-3 cEt ASOs at concentrations ranging from 3 pM to 3 µM. Concentration curves are plotted for BRET ratios in the presence of each unconjugated ASO. TAAR5 ASO sequences and K_D's can be found in Table S4.
