# Supplementary material for: Development of a Quantitative BRET Affinity Assay for Nucleic Acid-Protein Interactions
Source: PLoS One. 2016 Aug 29;11(8):e0161930. doi: 10.1371/journal.pone.0161930 (PMC5003356; doi:10.1371/journal.pone.0161930)
Supplement: S4 Fig — P54nrb-NLuc (A), NLuc-NCL (B), and NLuc/RPL5 (C) fusion proteins were expressed by transient transfection in Hela cells. Cell lysates were prepared and the fusion protein immunopreciptated with an antibody to NLuc. For ASO NanoBRET using the IP’ed protein (red) or crude lysates (green), binding affinities were determined by incubating 106 RLU of fusion protein with a 3’ ALEXA 594 conjugated 5-10-5 cEt gap-mer ASO at the indicated concentrations. Concentration curves were plotted for BRET ratios using GraphPad PRISM software. For Nano BRET in intact cells, the HeLa cells expressing the fusion protein were seeded in 96-well plates at 5000 cells/well. 24 hours after the initiation of transfection, cells were permeabilzed with 25–50 ng/mL digitonin in OptiMEM (blue) or PBS (blue dashed) plus the cEt gap-mer ASO at concentrations between 1 nM and 1 uM. After a 30 minute incubation NLuc substrate was added and BRET ratios determined as above. For thr RPL5 fusion, cells were also treated in OptiMEM in the presence of 0.2% saponin (black) or in OptiMEM with no permeabilizing agent (brown). (PDF) [file pone.0161930.s004.pdf]

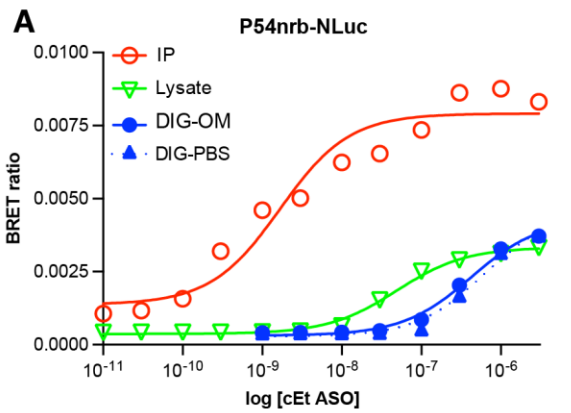

	IP	Lys	DIG-OM	DIG-PBS
K_D	1.667 ± 0.56	49.11 ± 6.08	424.8 ± 76.6	638.9 ± 158.4

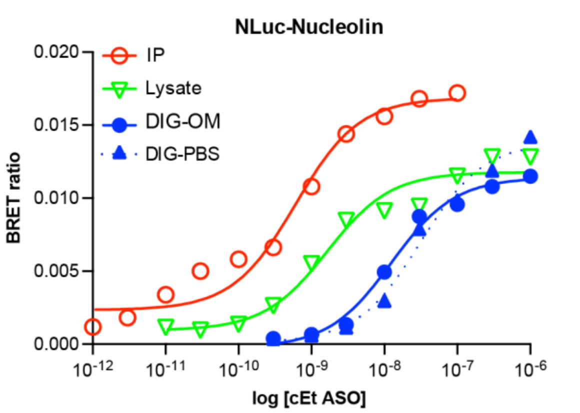

	IP	Lys	DIG-OM	DIG-PBS
K_D	0.607 ± 0.15	1.645 ± 0.42	12.45 ± 2.33	31.11 ± 6.68

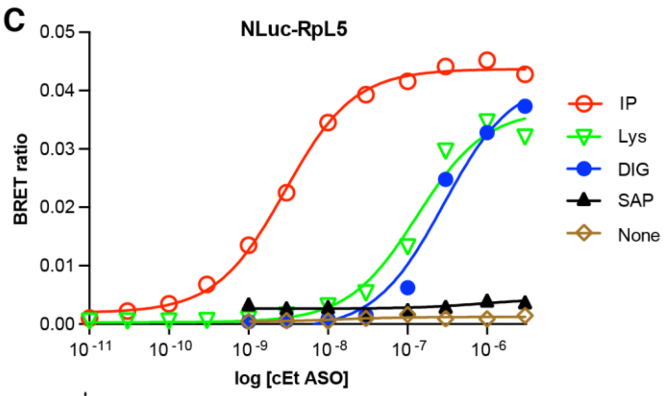

	IP	Lys	DIG	SAP	None
K_D	2.88 ± 0.23	131.7 ± 27.7	283.8 ± 74.7	>3000	>3000

Figure S4. ASO NanoBRET in permeabilized cells. P54nrb/NLuc (A), NLuc/NCL **(B)**, and NLuc/RPL5 **(C)** fusion proteins were expressed by transient transfection in Hela cells. Cell lysates were prepared and the fusion protein immunopreciptated with an antibody to NLuc. For ASO NanoBRET using the IP'ed protein (red) or crude lysates (green), binding affinities were determined by incubating 10^6 RLU of fusion protein with a 3' ALEXA 594 conjugated 5-10-5 cEt gap-mer ASO at the indicated concentrations. Concentration curves were plotted for BRET ratios using GraphPad PRISM software. For Nano BRET in intact cells, the HeLa cells expressing the fusion protein were seeded in 96-well plates at 5000 cells/well. 24 hours after the initiation of transfection, cells were permeabilzed with 25-50 ng/mL digitonin in OptiMEM (blue) or PBS (blue dashed) plus the cEt gap-mer ASO at concentrations between 1 nM and 1 uM. After a 30 minute incubation NLuc substrate was added and BRET ratios determined as above. For thr RPL5 fusion, cells were also treated in OptiMEM in the presence of 0.2% saponin (black) or in OptiMEM with no permeabilizing agent (brown).
