# Supplementary material for: Development of a Quantitative BRET Affinity Assay for Nucleic Acid-Protein Interactions
Source: PLoS One. 2016 Aug 29;11(8):e0161930. doi: 10.1371/journal.pone.0161930 (PMC5003356; doi:10.1371/journal.pone.0161930)
Supplement: S5 Fig — A) P54nrb/Nluc fusion protein was expressed by transient transfection of 3 different plates of Hela cells. Cell lysates were prepared and the fusion proteins immunoprecipitated with an antibody to P54nrb. Binding affinities were determined in separate experiments by incubating 106 RLU of fusion protein with a 3’ ALEXA 594 conjugated cEt gap-mer ASO at concentrations ranging from 10 pM to 3 uM. Binding curves are shown for each of the biological replicates and as well as the mean ± SEM for the 3 samples (black). (PDF) [file pone.0161930.s005.pdf]

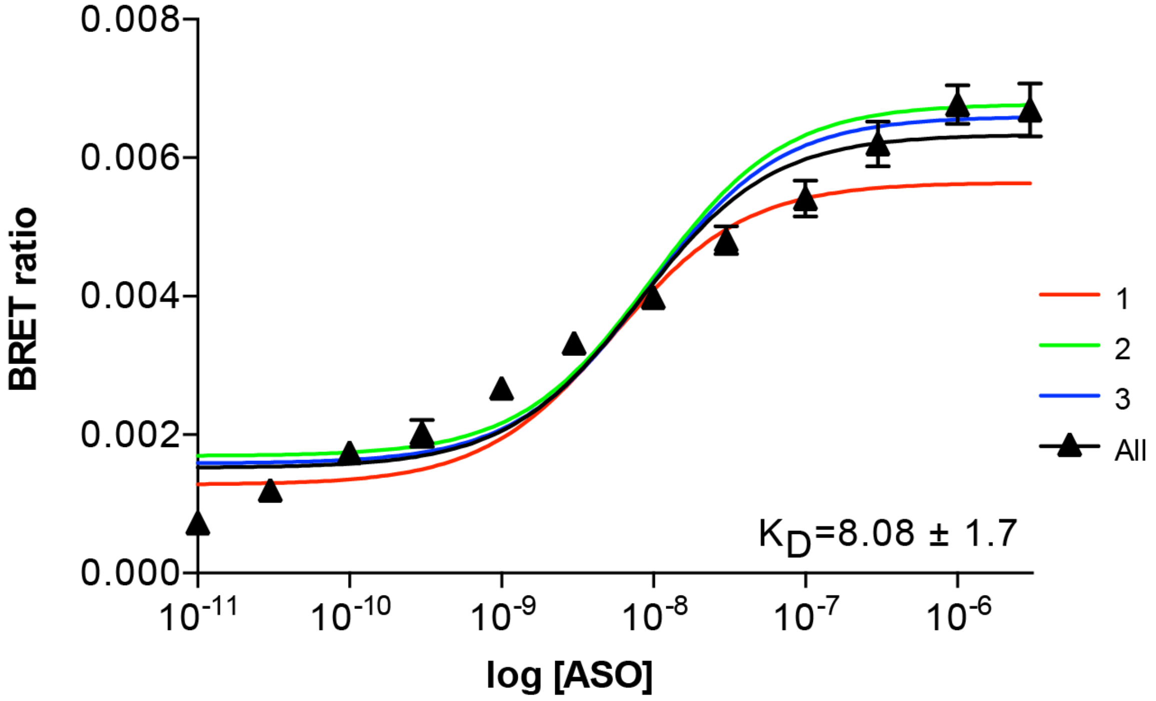

S5 Fig. ASO NanoBRET reproducibility. **A)** P54nrb/Nluc fusion protein was expressed by transient transfection of 3 different plates of Hela cells. Cell lysates were prepared and the fusion proteins immunoprecipitated with an antibody to P54nrb. Binding affinities were determined in separate experiments by incubating 10^6 RLU of fusion protein with a 3’ ALEXA 594 conjugated cEt gap-mer ASO at concentrations ranging from 10 pM to 3 uM. Binding curves are shown for each of the biological replicates and as well as the mean ± SEM for the 3 samples (black).
