# Supplementary material for: Development of a Quantitative BRET Affinity Assay for Nucleic Acid-Protein Interactions
Source: PLoS One. 2016 Aug 29;11(8):e0161930. doi: 10.1371/journal.pone.0161930 (PMC5003356; doi:10.1371/journal.pone.0161930)
Supplement: S1 Table — For gap-mer ASOs, 2’ modified bases (2’-alpha-flouro, (S)-cEt, and MOE) are indicated by bold type. The Ionis numbers of the gap-mer ASO are in parentheses. For AP1 oligonucleotides, the AP1 consensus site is underlined and consensus binding site mutations are indicated in red. (PDF) [file pone.0161930.s006.pdf]

ASO	Sequence	Chemistry	Fluorophore	site
5’ MOE (766633)	**CTGCT**AGCCTCTGGA**TTTGA**	PS MOE gap-mer	5' Alexa Fluor 594	PTEN
3’ MOE (766634)	**CTGCT**AGCCTCTGGA**TTTGA**	PS MOE gap-mer	3' Alexa Fluor 594	PTEN
5’ cEt (766635)	**CTGCT**AGCCTCTGGA**TTTGA**	PS cEt gap-mer	5' Alexa Fluor 594	PTEN
3’ cEt (766636)	**CTGCT**AGCCTCTGGA**TTTGA**	PS cEt gap-mer	3' Alexa Fluor 594	PTEN
5’ 2’-F (766637)	**CTGCT**AGCCTCTGGA**TTTGA**	PS 2’F gap-mer	5' Alexa Fluor 594	PTEN
3’ 2’-F (766638)	**CTGCT**AGCCTCTGGA**TTTGA**	PS 2’F gap-mer	3' Alexa Fluor 594	PTEN
RNase H1	CTGCTAGCCTCTGGATTTGA	uniform PO, DNA	3' Alexa Fluor 594	PTEN
RNase H1 comp	TCAAATCCAGAGGCTAGCAG	uniform PO, DNA	none	
RNase H1 comp	UCAAAUCCAGAGGCUAGCAG	uniform PO, RNA	none	
STAU1	CUGCUAGCCUCUGGAUUUGA	uniform PO, RNA	3' Alexa Fluor 594	PTEN
STAU1 comp	UAUGGGGUCAAAUCCAGAGGC<br>UAGCAGAAGGGAGUAACUA	uniform PO, RNA	none	
STAU1 comp	TATGGGGTCAAATCCAGAGGC<br>TAGCAGAAGGGAGTAACTA	uniform PO, DNA	none	
STAU1	CTGCTAGCCTCTGGATTTGA	uniform PO, DNA	3' Alexa Fluor 594	PTEN
AP1	CGCTTGA<u>TGAGTCA</u>GCCGGAA	uniform PO, DNA	3' Alexa Fluor 594	AP1
AP1 comp	TTCCGGCTGACTCATCAAGCG	uniform PO, DNA	none	AP1
AP1-M	CGCTTGA<u>TGACTTG</u>GCCGGAA	uniform PO, DNA	3' Alexa Fluor 594	AP1
AP1-M comp	TTCCGGCCAAGTCATCAAGCG	uniform PO, DNA	none	AP1

Table S1. ASOs used in study. For gap-mer ASOs, 2’ modified bases (2’-alpha-flouro, (S)-cEt, and MOE) are indicated by bold type. The Ionis numbers of the gap-mer ASO are in parentheses. For AP1 oligonucleotides, the AP1 consensus site is underlined and consensus binding site mutations are indicated in red.
