# Supplementary material for: Development of a Quantitative BRET Affinity Assay for Nucleic Acid-Protein Interactions
Source: PLoS One. 2016 Aug 29;11(8):e0161930. doi: 10.1371/journal.pone.0161930 (PMC5003356; doi:10.1371/journal.pone.0161930)
Supplement: S2 Table — (PDF) [file pone.0161930.s007.pdf]

Gene	Origene cDNA	vector	FP	RP	notes
hP54nrb	RC206688	pFN31K	gcat tcga C TCGAG c CAG AGT AAT AAA ACT TTT AAC TTG GAG	TCG AAT GCG AAT TCC TAG TAT CGG CGA CGT TTG TTT GG	
hP54nrb	RC206688	pFC32K	gcat tcga gctagc AGC CAC CATG g AGA GTA ATA AAA CTT TTA ACT TGG AGA	TCG AAT GCC TCG AGT CGT ATC GGC GAC GTT TGT TTG G	
Nmp1	RC203841	pFN31K	gcat tcga C TCGAG c ATG GAA GAT TCG ATG GAC ATG G	TCG AAT GCG AAT TCC TAT CAA TGC GCT TTT TCT ATA CTT GC	
Nmp1	RC203841	pFC32K	gcat tcga gctagc AGC CAC C ATG GAA GAT TCG ATG GAC ATG G	TCG AAT GCC TCG AGT CAT GCG CTT TTT CTA TAC TTG CTT G	
La	RC238025	pFN31K	gcat tcga C TCGAG c GCT GAA AAT GGT GAT AAT GAA AAG A	TCG AAT GCG AAT TCC TAC TAC TGG TCT CCA GCA CCA T	
La	RC238025	pFC32K	gcat tcga gctagc AGC CAC C ATG GCT GAA AAT GGT GAT AAT GAA AG	TCG AAT GCC TCG AGT CCT GGT CTC CAG CAC CAT TTT C	
Ku70	SC110901	pFC32K	gcat tcga gctagc AGC CAC C ATG TCA GGG TGG GAG TCA TAT	TCG AAT GCC TCG AGT CGT CCT GGA AGT GCT TGG TG	
RNASE H1	RC200595	pFN31K	gcat tcga C TCGAG c AGC TGG CTT CTG TTC CTG G	TCG AAT GCG AAT TCC TAT CAG TCT TCC GAT TGT TTA GCT C	
RNASE H1	RC200595	pFC32K	gcat tcga gctagc AGC CAC C ATG AGC TGG CTT CTG TTC CTG	TCG AAT GCC TCG AGT CGT CTT CCG ATT GTT TAG CTC CT	
STAU1	RC210564	pFC32K	gcat tcga gctagc AGC CAC C ATG AAA CTT GGA AAA AAA CCA ATG TAT A	TCG AAT GCC TCG AGT CGC ACC TCC CAC ACA CAG AC	
RPL5	MR204113	pFN31K	gcat tcga C TCGAG c GGG TTT GTG AAA GTT GTC AAG AAT	TCG AAT GCG AAT TCC TAG CTT TCA GCA GCC CTT TCC T	
RPL11	RC204006	pFN31K	gcat tcga C TCGAG c GCG GAT CAA GGT GAA AAG GAG	TCG AAT GCG AAT TCC TAT TTG CCA GGA AGG ATG ATC CC	
FUS	MR208306	pFC32K	gcat tcga gctagc AGC CAC C ATG GCT TCA AAC GAC TAT ACC C	TCG AAT GCC TCG AGT CAT ATG GCC TCT CCC TGC AGT	
NUCLEOLIN	MC203667	pFN31K	gcat tcga C TCGAG c GTA GAA GGC TCA GAA CCA ACT	TCG AAT GCG AAT TCC TAA CCA CCA AAG CCA CCT TCA C	RBDs only
NUCLEOLIN	MC203667	pFC32K	gcat tcga gctagc AGC CAC C ATG GTA GAA GGC TCA GAA CCA ACT	TCG AAT GCC TCG AGT CAC CAC CAA AGC CAC CTT CAC	RBDs only
PC4	RC204999	pFN31K	gcat tcga C TCGAG c CCT AAA TCA AAG GAA CTT GTT TCT TC	TCG AAT GCG AAT TCC TAC AGT TTT CTT ACT GCA TCA TCA ATG	
TCP1-beta	MR208575	pFN31K	gcat tcga C TCGAG c GCT TCC CTT TCC CTC GCA C	TCG AAT GCG AAT TCC TAA CAG GGG TGG TGA TCG GG	
ACTB	MR225133	pFN31K	gcat tcga C TCGAG c GAT GAC GAT ATC GCT GCG CT	ATG CGA ATT CCT AGA AGC ACT TGC GGT GCA CG	
HSP90	RC212496	pFC32K	gcat tcga gctagc AGC CAC C ATG GCT GAA GAA AAG GAA GAC AAA GAA	TCG AAT GCC TCG AGT CTG CTG CCA TGT AAC CCA TTG TT	mid domain only
ANXA2	MR205064	pFC32K	gcat tcga gctagc AGC CAC C ATG TCT ACT GTT CAC GAA ATC CTG	TCG AAT GCC TCG AGT CGT CAT CTC CAC CAC ACA GGT	
C-JUN	MR227043	pFN31K	gcat tcga C TCGAG c ACT GCA AAG ATG GAA ACG ACC T	ATG CGA ATT CCT AAA ACG TTT GCA ACT GCT GCG TT	
LRPPRC	SC120536	pFC32K	gcat tcga gctagc AGC CAC C ATG GAA CCT GAT TTC CAG AAA GAT ATA TT	TCG AAT GCC TCG AGT CAG AAG AGT TTT CCC TCA ATT TTC TTA	RBD
SFPQ	SC127926	pFC32K	gcat tcga gctagc AGC CAC C ATG TCT CGG GAT CGG TTC CG	TCG AAT GCC TCG AGT CGC CCA TTC GGC TGT AAC TTT C	

Table S2. Sequences of PCR primers used to generate cDNAs for directional in-frame cloning with NLuc.
