# Supplementary material for: Development of a Quantitative BRET Affinity Assay for Nucleic Acid-Protein Interactions
Source: PLoS One. 2016 Aug 29;11(8):e0161930. doi: 10.1371/journal.pone.0161930 (PMC5003356; doi:10.1371/journal.pone.0161930)
Supplement: S3 Table — (PDF) [file pone.0161930.s008.pdf]

gene	Deleted region	sense primer	antisense primer
hP54nrb	RRM1	CCCAACGAAGCCGTGTGCGCTTTGCCTG	CAGGCAAAGCGCACACGGCTTCGTTGGG
hP54nrb	RRM2	TCAGTATGTGTCCAACCCTGTGACTGTGGAGC	GCTCCACAGTCACAGGGTTGGACACATACTGA
La	LaM	GGCTGCCCTGGAGGCCGATGAGTATAAAAATGA	TCATTTTTATACTCATCGGCCTCCAGGGCAGCC
La	RRM1	CCTGAAGTGACTGATGAGTATAAAAATGATGTACTGCTAATACTTTTCAAGG	CCTTGAAAAGTATTAGCAGTACATCATTTTTATACTCATCAGTCACTTCAGG
La	RRM2	GAAAAGATTGGATGCTTGCTGAAAATAATAGAAGACCAACAAGAATCC	GGATTCTTGTTGGTCTTCTATTATTTTCAGCAAGCATCCAATCTTTTC
La	RRM1/2	TGACTGATGAGTATAAAAATGATGTAATAATAGAAGACCAACAAGAATCCC	GGGATTCTTGTTGGTCTTCTATTATTACATCATTTTTATACTCATCAGTCA
RNAse H1	HBD	GGCGGGCTCGAGCGTCAGGAAATCTGC	GCAGATTTCCTGACGCTCGAGCCCGCC

Table S3. Sequences of SDM primers used to generate deletion mutants for p54nrb, La, and RNAse H1.
