# Supplementary material for: Development of a Quantitative BRET Affinity Assay for Nucleic Acid-Protein Interactions
Source: PLoS One. 2016 Aug 29;11(8):e0161930. doi: 10.1371/journal.pone.0161930 (PMC5003356; doi:10.1371/journal.pone.0161930)
Supplement: S4 Table — 20,000 MEF cells were treated with 3.5 uM ASO by electroporation in 96 well pltes in a total volume of 100 ul. 24 hours later total RNA was purified and levels of TAAR5 mRNA accessed by qRT/PCR as described previously [11]. qRT/PCR FP: TGCTACCAGGTGAATGGGTCTT, RP: TGCGCAGGCCAGATAGATG, probe: AGGACAGTCCACCCGCTGGCC. For each ASO data is presented as the percent of mock treated control for 3 replicates. KD’s (nM) for NanoBRET ASO binding were determind us GraphPad PRISM software. (PDF) [file pone.0161930.s009.pdf]

IonisNo	Sequence	% Control Target	Std Dev	KD (LLRPPRC)	KD (NCL)	KD (H1)	KD (P54nrb)
660992	ACCCTGCCACGATGTA	5.94	0.00	0.01 ± 0	2.88 ± 0.52	46.3 ± 6.9	15.19 ± 0.73
661073	GTTAAGAAGGCTGTCC	18.82	14.57	0.16 ± 0.01	13.41 ± 2.33	149.3 ± 10.18	95.54 ± 12.16
660965	TCCACAGAGCGGACTG	22.40	18.87	1.20 ± 0.04	28.16 ± 7.77	189.1 ± 36.52	190.6 ± 47.12
660956	AACATGTCTGCCAGGG	27.33	0.82	0.42 ± 0.04	2.35 ± 0.21	10.52 ± 1.76	0.71 ± 0.27
661121	CCCTCCCTCCCGCTAG	27.77	27.01	0.22 ± 0	0.82 ± 0.08	20.45 ± 0.73	3.39 ± 0.84
661115	TCAGTCATGGTATAAA	38.17	7.62	0.18 ± 0.01	3.01 ± 0.25	51.11 ± 4.69	15.22 ± 1.49
661010	CTTCTAGCCACTGGCT	49.55	0.00	1.21 ± 0.04	18.99 ± 0.94	113 ± 13.47	42.69 ± 4.17
661103	TGAGAAGATCTCCCGG	59.91	34.12	0.19 ± 0.01	2.25 ± 0.10	25.35 ± 3.54	15.67 ± 3.01
660935	CCACTGCGCAGGCCAG	100.02	121.45	1.44 ± 0.05	12.28 ± 2.12	119.2 ± 21.97	74.83 ± 13.02
660899	CTGGGAACTGGTCACC	106.60	10.46	0.29 ± 0.01	3.95 ± 0.86	52.91 ± 8.56	15.36 ± 3.63
660908	GGGAGGAGGACAGCTC	110.74	89.61	0.30 ± 0.07	1.64 ± 0.34	1.63 ± 0.12	0.51 ± 0
660923	CCAGCGGGTGGACTGT	115.33	1.68	0.27 ± 0.01	7.37 ± 3.07	7.112 ± 2.93	1.22 ± 0.37

Table S4. TAAR5 ASOs activity and protein affinity. 20,000 MEF cells were treated with 3.5 uM ASO by electroporation in 96 well pltes in a total volume of 100 ul. 24 hours later total RNA was purified and levels of TAAR5 mRNA accessed by qRT/PCR as described previously [Vickers, 2015 #24835]. qRT/PCR FP: TGCTACCAGGTGAATGGGTCTT, RP: TGCGCAGGCCAGATAGATG , probe: AGGACAGTCCACCCGCTGGCC. For each ASO data is presented as the percent of mock treated control for 3 replicates. K_D's (nM) for NanoBRET ASO binding were determind us GraphPad PRISM software.
